# Pressing matter: why are ionic liquids so viscous?†

**DOI:** 10.1039/d1sc06857a

**Published:** 2022-02-08

**Authors:** Frederik Philippi, Daniel Rauber, Kira Lieberkind Eliasen, Nathalie Bouscharain, Kristine Niss, Christopher W. M. Kay, Tom Welton

**Affiliations:** Department of Chemistry, Molecular Sciences Research Hub, Imperial College London White City Campus London W12 0BZ UK t.welton@imperial.ac.uk; Department of Chemistry, Saarland University Campus B2.2 Saarbrücken Germany; “Glass and Time”, IMFUFA, Department of Science and Environment, Roskilde University P.O. Box 260 DK-4000 Roskilde Denmark; University of Lyon, INSA Lyon, CNRS, LaMCoS, UMR 5259 69621 Villeurbanne France; London Centre for Nanotechnology, University College London 17-19 Gordon Street London WC1H 0AH UK

## Abstract

Room temperature ionic liquids are considered to have huge potential for practical applications such as batteries. However, their high viscosity presents a significant challenge to their use changing from niche to ubiquitous. The modelling and prediction of viscosity in ionic liquids is the subject of an ongoing debate involving two competing hypotheses: molecular and local mechanisms *versus* collective and long-range mechanisms. To distinguish between these two theories, we compared an ionic liquid with its uncharged, isoelectronic, isostructural molecular mimic. We measured the viscosity of the molecular mimic at high pressure to emulate the high densities in ionic liquids, which result from the Coulomb interactions in the latter. We were thus able to reveal that the relative contributions of coulombic compaction and the charge network interactions are of similar magnitude. We therefore suggest that the optimisation of the viscosity in room temperature ionic liquids must follow a dual approach.

## Abstract 2

We use an experimental approach to compare an ionic liquid with a molecular mimic, focusing on viscosities. Charge network and coulombic compaction contribute significantly to the high viscosity of ionic liquids; we discuss the implications on their design and optimisation.

## Graphical abstract

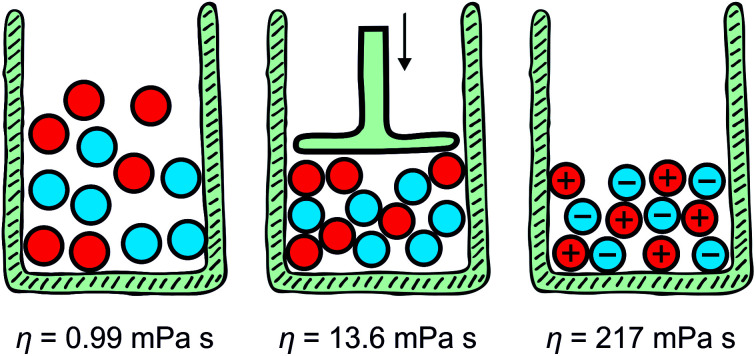

## Introduction

In recent years, ionic liquids have transformed from a scientific curiosity to extensively used functional fluids, both in academia and industry.^1–5^ However, the practical applicability of most ionic liquids is limited by their high viscosity compared with conventional molecular solvents. This is a key aspect for applications such as batteries, gas separation or biomass processing. In order to optimise the viscosity, it is necessary to develop a mechanistic understanding of the difference between how viscosity arises in ionic liquids and conventional molecular solvents.

**Fig. 1:**
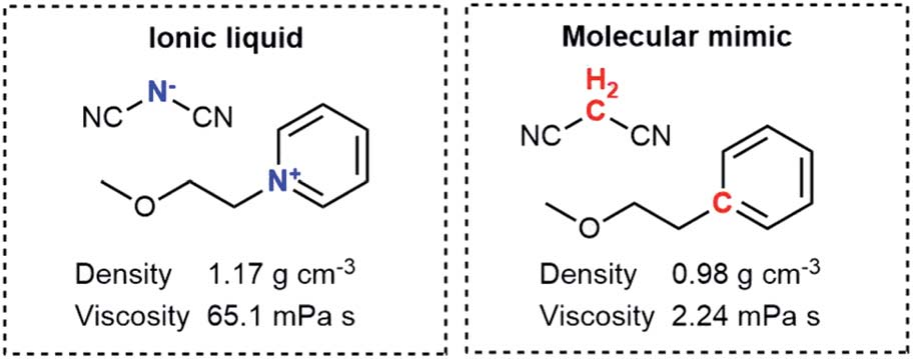
The ionic liquid (left) and molecular mimic (right) investigated by Shirota and Castner.^9^

A fair, unbiased comparison between ionic liquids and conventional molecular solvents necessitates two systems which are as similar as possible; one charged, and one neutral. The neutral system has been called the ‘molecular mimic’^6,7^ and is a mixture of neutral analogues of the anionic and cationic molecular constituents.^6–8^ To ensure similarity, the molecular mimic and the corresponding ionic liquid should ideally be isoelectronic and isostructural to each other. Fig. 1 shows the molecular mimic used by Shirota and Castner, together with the viscosity and density values at room temperature and ambient pressure.^9^ Crucially, the viscosity of the ionic liquid is almost 30 times that of the molecular mimic, despite the similar molecular structures. One might interpret this factor of 30 as the difference between conventional molecular solvent and ionic liquid, *i.e.* the isolated effect of the added charge. However, the ionic liquid also has a higher density than the molecular mimic, which still constitutes a bias. The higher density is the result of coulombic compaction, *i.e.* strong (attractive) coulombic interactions which reduce the volume of the liquid phase.^9^

The differences in density and viscosity lead to an important question: what is the degree to which coulombic compaction causes the high viscosity of ionic liquids? It is well known that an increase in density (*i.e.* pressure) generally leads to an increase in viscosity.^10,11^ Indeed, the viscosity of both molecular and ionic liquids can often be expressed as a function of *ρ*^*γ*^/*T*, where *ρ* is the density, *T* the temperature and *γ* a material parameter.^12–15^ Consequently, the comparison between a molecular mimic and an ionic liquid should be made under isodensity conditions. In other words, the molecular mimic must be subjected to pressure high enough so that its density becomes equal to that of the ionic liquid under ambient pressure at the same temperature. It is conceivable that the two systems would then also have similar viscosities.^16^ If this were the case, it would imply that the high density itself was the cause of the high viscosity.

A wide variety of models with considerable conceptual overlap have been developed to describe viscous flow of ionic liquids.^17–33^ Some models aim to further our understanding of the underlying physics from a basic scientific viewpoint with often limited predictive value. Other models aim to provide predictive tools – which are numerically accurate but often without physical basis – for engineering purposes. At present, there is considerable dispute about which of the approaches is preferable for ionic liquids, but clearly the ultimate goal is to develop physically sound models which also provide reliable quantitative predictions.

In order to resolve these difficulties, we separate the change in viscosity into two steps. First from the molecular mimic under ambient conditions to the molecular mimic when isodense with the ionic liquid, and second from the isodense molecular mimic to the ionic liquid. The viscosity models can then be separated into two groups accordingly.

Some researchers favour models describing the indirect effects of coulombic compaction, for example hole theory, free volume theory or the shoving model.^24,28–32,34^ These models are not unique to ionic liquids, but gain significance due to the apparent ‘high pressure’ conditions. Mechanistically, the processes are dominated by molecular level relaxation. Related concepts such as the ion cage or activation volumes are commonly used in the ionic liquids community.^35–38^

Others prefer models which approach the problem on a collective rather than molecular level. In contrast to molecular liquids, ionic liquids are subject to intermolecular electroneutrality conditions, leading to the formation of a charge network.^39,40^ Viscous flow was found to be coupled to the structural relaxation of this charge network.^19–21^ Shelepova *et al.* recently compared the structure of an ionic liquid and its isodense molecular mimic by means of molecular dynamics simulations.^41^ The authors observed rather similar total radial distribution functions for the charged and uncharged systems, despite the additional screening conditions for the charged system which lead to the formation of a charge network, *i.e.* a shell structure of oppositely charged ions around each reference ion.^41–43^

The concept of experimentally measuring molecular mimics under isodensity conditions was explored in 1968 by Morrison and Lind,^44^ however only at elevated temperatures.^45,46^ For tetrabutylammonium tetrabutylborate [NBu_4_][BBu_4_], a viscosity of 18.7 mPa s at 114 °C was reported. The viscosity of the corresponding molecular mimic, tetrabutylmethane [CBu_4_][CBu_4_], was about 2.5 mPa s at isodensity conditions and 0.92 mPa s at ambient pressure (see ESI, Section 1†).^44^ Hence, the two factors by which viscosity increases were 2.7 (from the molecular mimic to the isodense molecular mimic) and 7.5 (from the isodense molecular mimic to the ionic liquid).^44^ Data at lower temperatures are not available due to the high melting point of [NBu_4_][BBu_4_] of about 110 °C.^45^ However, comparison with the viscosity ratios at higher temperatures shows that the relative difference in viscosity between the ionic liquid and the isodense molecular mimic increases significantly at lower temperatures. At 163 °C, the viscosity ratios were 2.4 (from the molecular mimic to the isodense molecular mimic) and 5.1 (from the isodense molecular mimic to the ionic liquid).

Molecular dynamics simulations can also be employed to directly compare ionic liquids and their molecular mimics. Roy *et al.* performed molecular dynamics simulations on a coarse grained model of 1-butyl-3-methylimidazolium hexafluorophosphate.^47^ In order to realise the molecular mimic, the authors simply removed electrostatic interactions. At a simulation temperature of 450 K, the viscosity increases by a factor of 75 from the isodense molecular mimic (1.1 mPa s) to the ionic liquid (83 mPa s).^47^ Unfortunately a comparison between the molecular mimic under ambient pressure and the isodense molecular mimic is not feasible since at this temperature the molecular mimic is a gas (under ambient pressure).

The large difference between isodense molecular mimics and ionic liquids is surprising, given that Shirota *et al.* observed only a factor of 30 between their molecular mimic under ambient conditions and the ionic liquid. Critically, if the experimental results from Morrison and Lind were universally transferable, then the relative viscosity increases observed by Roy *et al.* should be smaller than those observed by Shirota *et al.* since the latter are (a) at a lower temperature and (b) additionally include the change from the molecular mimic under ambient pressure to the molecular mimic under isodensity conditions. Importantly, Roy *et al.* revised their model to more closely match experimental values.^48^ In the revised model, the viscosity of the ionic liquid is only 3.4 mPa s at 450 K,^48^ compared to 83 mPa s in the original model.^47^ A direct comparison at 450 K across the two models would not be meaningful due to various changes to simulation conditions and the force field itself. However, the authors provide the required data at 350 K: here, the viscosity changes by a factor of 22 from the isodense molecular mimic (1.2 mPa s) to the ionic liquid (26 mPa s).^48^ In addition, the authors provide viscosity data under ambient pressure and temperature. At 298 K, the molecular mimic has a viscosity of 0.42 mPa s, compared to the ionic liquid with a viscosity of 330 mPa s (with a reported uncertainty of ±100 mPa s).^48^ This corresponds to a change in viscosity by a factor of 785, however there is no information regarding the relative contributions of charge network and coulombic compaction.

A similar approach has been followed by Park *et al.* who performed MD simulations on a generalised coarse grained model based on ionic liquids such as 1-butyl-3-methylimidazolium hexafluorophosphate.^49^ The authors provide diffusion coefficients, from which the viscosity ratios can be estimated (see ESI Section 1†). The relative increases in viscosity from the isodense molecular mimic to the ionic liquid are 12 (at 370 K) and ≈370 (at 250 K). These results clearly show the pronounced temperature dependence of the viscosity difference between ionic liquids and their molecular mimics, even if the numerical values are not quantitatively transferable to realistic ionic liquids.

Due to recent advances in the field of (room temperature) ionic liquids, a study at ambient temperatures is now possible. Here, we demonstrate a proof of concept for the entire approach, from the selection of an experimental system to the characterisation under high pressure. Our results have far-reaching implications for the development of viscosity models, which must account for the diverse nature of ionic liquids.

## Choice of experimental system

The selection of an appropriate experimental system is non-trivial, since a multitude of requirements have to be simultaneously fulfilled:

(1) The molecular mimic should be structurally similar to the ionic liquid.

(2) Both molecular mimic and ionic liquid must be experimentally accessible.

(3) The two components of the molecular mimic must not react with each other, even under pressure.

(4) The components must be miscible, and the molecular mimic must remain homogeneous and liquid under isodensity conditions.

(5) The equipment must be resistant to the involved compounds.

The similarity of the ionic liquid and the molecular mimic is an obvious requirement, but some cases require compromises. For example, the malononitrile chosen by Shirota and Castner as a neutral analogue to the dicyanamide anion may introduce hydrogen bonding to the ether group present in the cation's neutral analogue.^9^ Hydrogen bonds between ether groups and the C–H acidic malononitrile have been reported in the literature using both experimental and theoretical methods.^50–53^ An alternative would be cyanogen oxide O(CN)_2_, but this violates at least the second and third requirements. Similarly, one trivial neutral analogue of the acetate anion is the rather hazardous and volatile acetyl fluoride. For our preliminary studies, therefore, we considered acetyl chloride, acetic acid and nitromethane as neutral analogues.

The second point applies for compounds such as Ar, CF_4_ and SF_6_, which are the trivial neutral analogues for Cl^−^, [BF_4_]^−^ and [PF_6_]^−^. These neutral analogues are, while stable, all gaseous under ambient conditions, presenting a significant obstacle for experimental investigations. In contrast, neutral analogues of this type are predestined for computer simulation. Even if the experimental setup can be prepared with an equimolar mixture of, say, propane and tetrafluoromethane, it would be preferable to have an estimate for required pressures and liquid–liquid critical points beforehand.

We initially considered the commonly used ionic liquid [C_4_C_1_im][NTf_2_]. To this end, the imidazolium cation could be replaced with a furan or pyrrole based neutral analogue, and the anion could be replaced with triflic anhydride OTf_2_, the free acid HNTf_2_ or methylene ditriflone CH_2_Tf_2_. However, as these compounds are rather reactive, they are not compatible with each other and indeed many other neutral analogues. Phosphonium and ammonium ionic liquids are much more benign in this respect, as they may be mimicked by thermodynamically stable silanes and hydrocarbons.

The fourth point must be kept in mind for the measurements under high pressure. For example, we considered nitromethane as a neutral analogue for the acetate anion. At ambient temperature, pure nitromethane solidifies at 400 MPa, even if the decomposition pressure itself is too high (around 30 GPa) to be of relevance for the measurements in this work.^54,55^ Similar issues arise for the actual molecular mimic, *i.e.* the mixture of two neutral analogues. High pressure might effectively raise the critical temperature of this mixture to above ambient temperatures, leading to undesirable phase separation.^56^ Our solution to this problem was to optimise towards molecular mimics with low critical temperatures. To this end, promising molecular mimics were subjected to progressively lower temperatures (ambient temperature 20 °C, fridge 5 °C, freezer −20 °C, dry ice −78 °C). For example, the molecular mimic composed of triethyl pentyl silane and 1-nitropropane was miscible at 20 °C, but not at 5 °C. A similar molecular mimic with 1-nitrohexane instead of 1-nitropropane remained homogeneous and liquid at −20 °C, but solidified at −78 °C.

Finally, the equipment itself puts restrictions on the scope of molecular mimics that can be investigated. Many instruments for high-pressure rheology and densitometry are built for engineering fluids, without consideration of resistance to aggressive compounds.

**Fig. 2:**
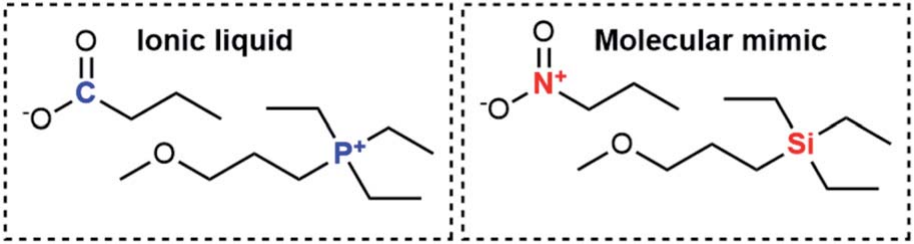
The ionic liquid/molecular mimic combination used in this work.

Considering all these points, we identified the experimental system shown in Fig. 2. The ionic liquid is triethyl(3-methoxypropyl)phosphonium butyrate, the corresponding molecular mimic is an equimolar mixture of triethyl(3-methoxypropyl)silane and 1-nitropropane. The presence of an ether group helped achieve miscibility over a wide temperature range. For the sake of simplicity, we will henceforth use “the ionic liquid” and “the molecular mimic” for this system. Details on the exploratory experiments leading to this choice can be found in the ESI, Section 2.†

## Results

The densities of the ionic liquid and the molecular mimic were measured at ambient pressure as a function of temperature (see ESI, Section 3†). The experimental densities at room temperature and ambient pressure were 0.874(1) g cm^−3^ for the molecular mimic and 1.032(1) g cm^−3^ for the ionic liquid.

Furthermore, we have measured the viscosity of the ionic liquid and the molecular mimic at ambient pressure (see ESI, Section 4†). The experimental viscosities at room temperature and ambient pressure were 0.86 mPa s for the molecular mimic and 217 mPa s for the ionic liquid. These values were obtained from Arrhenius and Vogel–Fulcher–Tammann fits, respectively, see ESI, Section 4.† However, our cone-plate setup was optimised for (relatively viscous) ionic liquids and does not perform well with low viscosity fluids. Hence, we repeated the measurement at ambient pressure and room temperature using a setup with coaxial geometry, and obtained a viscosity of 0.99(4) mPa s for the molecular mimic. While not significantly different from the cone-plate setup, this value is in quantitative agreement with the viscosity value obtained from the falling body experiment and will be used henceforth.

**Fig. 3:**
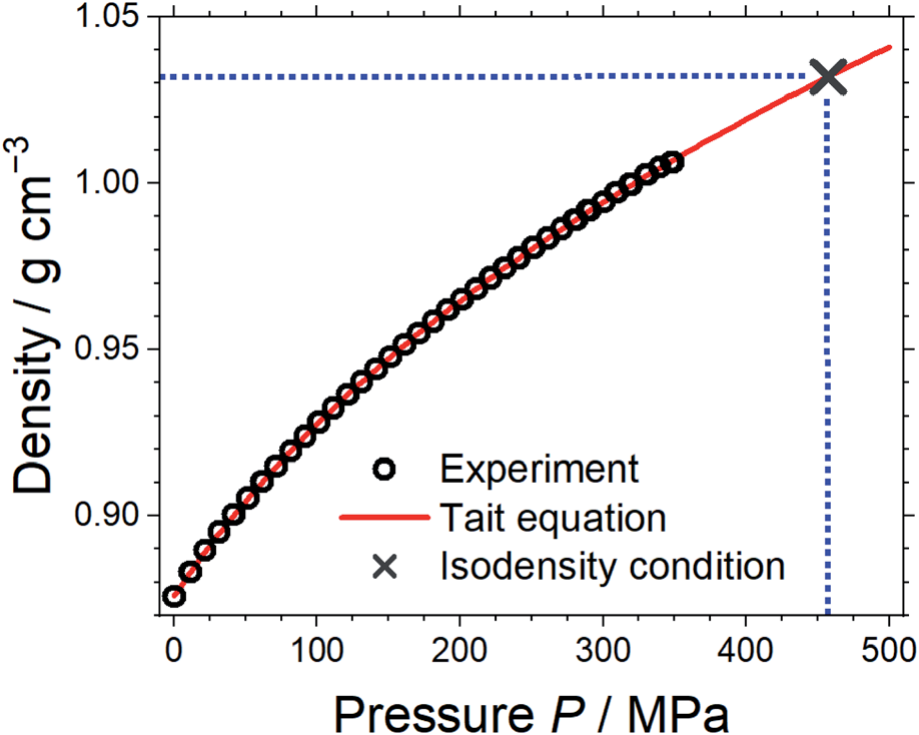
Density of the molecular mimic as a function of pressure. The Tait equation was used to extrapolate to the density of the corresponding ionic liquid, and the blue dashed lines indicate how the pressure required for isodensity conditions was obtained.

**Fig. 4:**
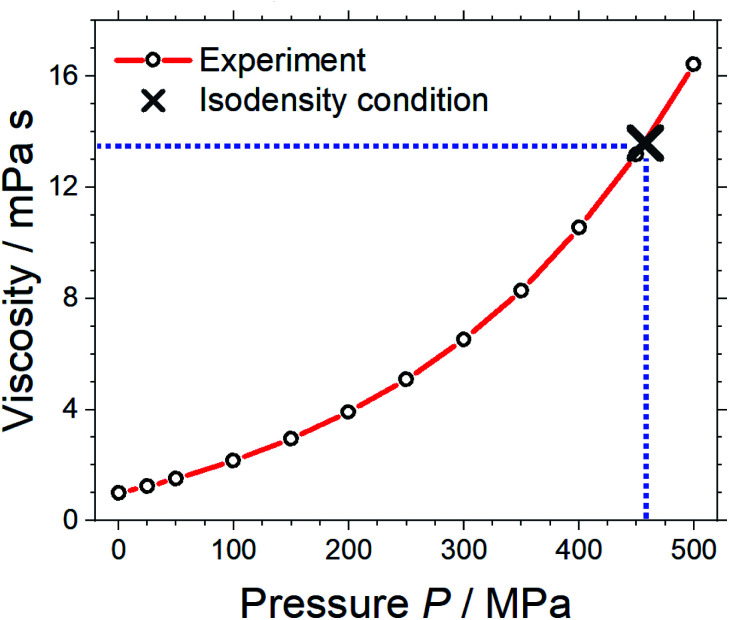
Viscosity of the molecular mimic as a function of density. The blue dashed lines indicate the viscosity under isodensity conditions.

Pressure–volume measurements were then performed to identify the pressure required for isodensity conditions, *i.e.* the pressure at which the density of the molecular mimic reaches 1.032 g cm^−3^. The density was extrapolated from the highest attainable pressure with our equipment (≈340 MPa) using the Tait equation.^57,58^ The results are presented in Fig. 3, details can be found in the ESI, Section 3.† We thus identified ≈460 MPa as the pressure for isodensity conditions. Subsequently, the viscosity of the molecular mimic was measured as a function of pressure up to 500 MPa using a falling body viscometer. The viscosity of the molecular mimic at a pressure of 460 MPa, *i.e.* at a density of 1.032 g cm^−3^, was approximately 14 mPa s, Fig. 4. Details of the high-pressure rheology and the interpolation can be found in the ESI, Section 4.†

In contrast to viscosity, diffusion coefficients can be determined separately for cation and anion. Diffusion coefficients were measured at ambient pressure and 25 °C, Table 1.

**Table 1:** Diffusion coefficients obtained from PFGSTE NMR diffusometry

System	Constituent	Diffusion coefficient
Molecular mimic	Si222(3O1)	8.46 × 10^−10^ m^2^ s^−1^
	Nitropropane	1.39 × 10^−9^ m^2^ s^−1^
Ionic liquid	[P222(3O1)]^+^	5.39 × 10^−12^ m^2^ s^−1^
	[C_3_H_7_COO]^−^	5.90 × 10^−12^ m^2^ s^−1^

## Discussion

**Fig. 5:**
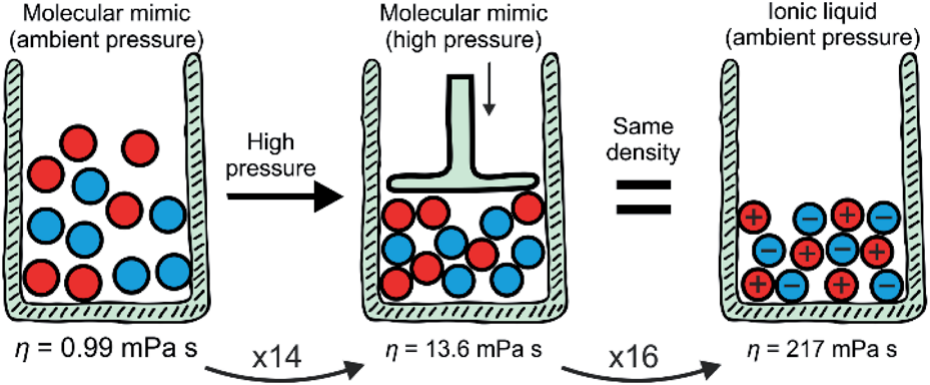
Summary of the results. Scheme adapted from ref. 8 – published by The Royal Society of Chemistry.

The key findings of this work are summarised schematically in Fig. 5. The viscosity of the ionic liquid at ambient temperature and pressure is higher by a factor of 219 than the viscosity of the isostructural, isoelectronic molecular mimic. This significant increase in viscosity can be separated into two contributions using pressure-dependent densitometry and rheology. First, the higher density of the ionic liquid – or, equivalently, the high pressure isodensity conditions for the molecular mimic – leads to an increase in viscosity. This can be understood in terms of viscosity models established for molecular liquids. Second, the charged nature of the ionic liquids leads to an additional increase in viscosity due to additional electrostatic restrictions on the motion of molecular ions.

Importantly, the relative contribution to the bulk viscosity was of equal magnitude in this case, with the viscosity increasing by a factor of 14 and 16. Hence, the indirect effects of coulombic compaction (factor of 14) and the direct effects of the charge network (factor of 16) are equally important. It appears that viscous flow in ionic liquids at room temperature enters a regime where a balance of different relaxation mechanisms becomes important, rather than just one dominating mechanism. This finding is interesting in light of the viscosity models discussed in the literature, many of which are reported to be applicable to ionic liquids.^17–23,25–33^ Indeed, the debate mentioned above is resolved insofar as different approaches to model the viscosity of ionic liquids seem to be not only justified, but necessary.

The two major contributions to ionic liquid viscosity are also important from a practical point of view. The results shown in Fig. 5 demonstrate that the optimisation of viscosity at room temperature must follow a dual approach. Different design concepts have been proposed to adjust the viscosity of ionic liquids, such as hydrogen bonding or conformational flexibility. For example, Fumino *et al.* proposed directional hydrogen bonding as a way to disrupt the charge network.^59^ This hypothesis could be tested using the approach we presented in this work. To name an example regarding the second contribution, *i.e.* Coulombic compaction, several groups have proposed conformational flexibility as a means to facilitate dynamics such as viscous flow and diffusion.^35,60^

The two major contributions observed for the ionic liquid in this work can be expected to be relevant for other ionic liquids as well, since coulombic compaction and the formation of a charge network will be present in any case. The central question for future work is how ionic liquids can be designed to shift the balance in one or the other direction.

Design concepts tend to mechanistically exploit one of the two major contributions. Hence, there is a limit to what can be achieved with, say, conformational flexibility. Lowering the viscosity of an ionic liquid below this limit will require additional leverage from complementary design concepts, for example the addition of molecular solvent to facilitate momentum transport without violating electroneutrality conditions. Both components must be considered during the design process; for example focusing on directional bonding will not give the best possible result if conformational flexibility is disregarded, and *vice versa*.

It is worth comparing the results of this work with an overview of the literature data. To this end, Table 2 provides a summary of the prior work presented in the Introduction.

**Table 2:** Summary of viscosity ratios comparing ionic liquids (IL), molecular mimics (MM) and molecular mimics under isodensity conditions (MM*)

Setup	Temperature	MM → MM*	MM* → IL	Overall (MM → IL)	References
Coarse grained MD simulation	450 K	—	75	—	47
Coarse grained MD simulation after refinement	350 K	—	22	—	48
	298 K	—	—	785^a^	48
Coarse grained MD simulation	370 K	—	12^b^	—	49
	250 K	—	Approx. 370^b^	—	49
Experimental, see Fig. 1	295 K	—	—	30^c^	9
Experimental	436 K	2.4	5.1	12	44
	387 K	2.7	7.5	20	44
Experimental	298 K	14	16	219	This work

a The factor is approximately 550 to 1000 within the viscosity uncertainty.

b Obtained *via* diffusion coefficients, see ESI Section 1.

c Likely too low due to additional hydrogen bonding in the molecular mimic.

The viscosity ratios from MD simulations cover a wide range, however unfortunately no comparison is possible between the three relevant viscosity ratios due to a lack of data. A comparison of relative values based on one type of simulation would be preferable since MD simulations rarely provide quantitative predictions.

The results from MD simulations are furthermore very sensitive to the simulation setup. For example, the viscosity of the ionic liquid in the refined model by Roy *et al.* is 3.4 mPa s at 450 K, compared to 83 mPa s in the original model.^47,48^ The refined model includes charge scaling, *i.e.* a mean-field version of polarisability and charge transfer.^48^ Phenomena such as polarisability are key for accurate simulations of ionic liquids.^61,62^ Error cancellation, otherwise a strength of relative comparisons from MD simulations, is less helpful if results are to be compared between charged and uncharged systems.

Our data and the data from Morrison and Lind both show a balance of the two contributions to viscosity, especially at room temperature. The overall viscosity increase reported by Shirota *et al.* is much lower than what we observed. This might be due to hydrogen bonding induced by the malononitrile.^50–53^ It would be desirable to have high pressure data for the molecular mimic proposed by Shirota *et al.* at hand. However, the molecular mimic is likely to solidify under high pressure since the melting point of malononitrile is near room temperature.

Diffusion coefficients, unlike the viscosity, can be measured separately for each constituent and thus give access to additional information. The diffusion coefficients can be compared by analogy to the viscosity values, *cf.* the Stokes–Einstein relation in the ESI, Section 1.† We were unable to access the high pressures required for isodensity conditions, however we have measured diffusion coefficients under ambient pressure and at 25 °C. The corresponding diffusion coefficient ratios between the molecular mimic and ionic liquid diffusion are 157 for the cation (analogue) and 236 for the anion (analogue). Thus, the overall increase in viscosity from the molecular mimic to the ionic liquid translates to a comparable decrease in the translational diffusion. Interestingly, the ratio of diffusion between anion and cation (analogues) decreases from *D*(nitropropane)/*D*(Si222(3O1)) = 1.64 in the molecular mimic to *D*([C_3_H_7_COO]^−^)/*D*([P222(3O1)]^+^) = 1.09 in the ionic liquid. Hence, the diffusion coefficients of the two constituents are much more similar in the ionic liquid than in the molecular mimic, which can be rationalised by the electrostatic interactions coupling the motion of cations and anions. The degree to which this coupling of motion already occurs under isodensity conditions, if at all, would be an interesting topic for future experimental and theoretical studies.

Overall, this study clearly shows how valuable insight can be gained from a comparison of ionic liquids and their molecular mimics. Without doubt, the practical importance of understanding the transport properties in ionic liquids justifies the considerable effort of performing experiments at such high pressures. It would be intriguing to study more types of ionic liquids in the future to probe the generality of the conclusions drawn from the phosphonium butyrate in this work.

Furthermore, during the selection process, we observed incompatibilities which in themselves are interesting. For example, in several cases, the cation and anion neutral analogues were not miscible. Naturally, in the ionic liquid, the two constituents must mix due to electroneutrality conditions. We hypothesise that this effect – known as nanosegregation – to some degree counteracts the effects of the charge network. The use of molecular mimics can help to identify such cases, using the mixing behaviour of the cation and anion neutral analogues as design element.

The future development of physically sound models should consider both coulombic compaction and the charge network as the two main contributors to ionic liquid viscosity. For example, mode coupling is a good approach to study and describe the connection between viscous flow and the charge network.^19,63,64^ Here, future work could explore the evolution of the dynamic structure factor of molecular mimics at wavenumbers corresponding to the charge network in the corresponding ionic liquid with the help of molecular dynamics simulations. The use of molecular dynamics has the added advantage that high pressures and even intermediates between molecular mimic and ionic liquid – with fractional charges on the molecules – are possible. *Vice versa*, approaches such as hole theory should factor in the presence of a charge network. In other words, a good model for any transport property in ionic liquids must be able to predict the difference between the isodense molecular mimic and the ionic liquid.

From an engineering point of view, the results from this work can be used to select appropriate models. Good examples are machine learning approaches, which are becoming more and more popular recently for quantitative predictions of ionic liquid properties.^65,66^ Machine learning algorithms use a number of input properties to produce an estimate of an output property. Easily accessible input properties, such as molecular weight or connectivity, are advantageous. Based on our observations, both coulombic compaction and charge network formation should be present in the input properties. Furthermore, it might be beneficial to introduce feature extraction with coulombic compaction and charge network formation as intermediate features to improve the performance of machine learning algorithms.

For future studies, it could also be worth expanding the measurements to several isotherms, which in combination with density scaling would allow the use of a ‘density–temperature superposition principle’, in analogy to the time–temperature superposition principle.^67,68^ This might enable the experimental comparison of those pairs of ionic liquid and molecular mimic where isodensity conditions are not attainable, but where common points of *ρ*^*γ*^/*T* can be found (see ESI, Section 1†). For example, it might not be possible to measure the experimental system shown in Fig. 1 under isodensity conditions due to pressure-induced crystallisation. In this case, common points of *ρ*^*γ*^/*T* could still be accessible by recording additional isotherms for the ionic liquid (at higher temperature) and molecular mimic (at lower temperature).

Finally, experimentally challenging systems such as CF_4_/[BF_4_]^−^ may still be studied *in silico* by means of molecular dynamics simulation. Here, one of the key issues remains consistency of the parameterisation across charged and uncharged systems, for example with regards to polarisability. Our experimental results at room temperature could be used to benchmark force fields and simulation setups. Once a reliable (classical simulation) model has been identified it can be used to generate further valuable data points at different temperatures or pressures, with considerably lower expense and hazards compared to the experimental high pressure studies.

## Methods

Samples were prepared under Schlenk conditions or in a glovebox, ionic liquids were dried in high vacuum before use. AgNO_3_ solution was used to confirm the absence of halides in the carboxylate ionic liquids. Syntheses and (additional) physicochemical characterisation are described in detail in the ESI.†

Densities as a function of temperature under ambient pressure were measured with a 5 mL (nominal) Reischauer pycnometer (Neubert Glas, Geschwenda, Germany) calibrated with octane and confirmed with water.^69^ The pycnometer was dried in vacuum before use, weighed, and filled with sample to above the mark in a glovebox. The pycnometer was then sealed, placed in a thermostat bath (thermostatted to ±0.01 K), equilibrated for 20 min, the liquid level adjusted to the mark with a Pasteur pipette, cooled to room temperature, and weighed again.

The density of the molecular mimic under high pressure was measured to find the isodensity conditions using a U111 high pressure pump and an MV1-30 pressure chamber, both provided by Unipress in Poland. The sample is loaded into a cylindrical container with a movable piston at one end and the container is then submerged into enclosed pressure fluid which is connected to a high-pressure pump. The increasing pressure moves the piston inwards, compressing the liquid sample, during which the displacement of the piston is recorded. The density change is then calculated using the absolute mass and the change in volume of the sample due to the increase in pressure. Measurements were performed from 0–350 MPa at 25 °C.

The viscosity under ambient pressure was measured with a cone-plate setup as well as with a coaxial setup. The cone-plate setup was used on a stress-controlled MCR 301 rheometer (Anton Paar, Graz, Austria) with a CP50-1 cone (diameter 49.95 mm, cone angle 1°) and a gap size of 0.101 mm. The measurements were performed under nitrogen atmosphere with shear rates varying from 5 to 150 s^−1^ in 30 linear steps. Newtonian behaviour was observed, and the viscosity obtained as an average over the shear rates. The temperature was allowed to equilibrate for 15 min before the measurements. The coaxial setup was used only for the molecular mimic with outer radius 14.4600 mm, inner radius 13.3292 mm, gap 1.1308 mm. The temperature was set to 25 °C with a Peltier heater.

Viscosity measurements under high pressure up to 500 MPa were performed at LaMCoS – INSA de Lyon using a falling body viscometer as described by Scott Bair.^70^ To this end, the sample is filled in a cartridge containing falling body (=sinker). One end of the cartridge is sealed with a plug, the other with a moveable piston to allow for pressure transfer. The cartridge is placed in a high pressure vessel surrounding by a pressurising medium. The high pressure vessel containing the cartridge is then rotated, causing the sinker to fall, the position of the latter is detected *via* a linear variable differential transformer. The viscosity is obtained from the falling time.

Diffusion coefficients were measured on an Avance Neo 500 MHz NMR spectrometer (Bruker, Billerica, USA) with Prodigy TCI CryoProbe (maximum gradient strength 65.7 G cm^−1^) using the pulsed field-gradient stimulated echo sequence with bipolar gradients and longitudinal eddy current delay (‘ledbpgp2s’ in the Bruker library) and smoothed rectangular gradient pulses similar to previous work.^71^ The individual self-diffusion coefficients of the constituents could be determined using resolved signals of the two species. The uncertainty of the measurement with this setup is approximately 2% of the absolute value (from our own repeat measurements on this setup and comparing different pulse sequences).

## Author contributions

Frederik Philippi: conceptualisation, methodology, investigation (syntheses of molecular mimics, mixing experiments), writing (original draft, revision), visualization, project administration. Daniel Rauber: investigation (synthesis of the ionic liquid, physicochemical characterisation). Kira Lieberkind Eliasen: validation, formal analysis (density fitting), investigation (pressure-dependent densitometry). Nathalie Bouscharain: validation, formal analysis (viscosity fitting), investigation (pressure-dependent rheology). Kristine Niss: resources (high pressure laboratory). Christopher W. M. Kay: resources (physicochemical characterisation equipment). Tom Welton: supervision, funding acquisition, resources (facilities for syntheses).

## Conflicts of interest

There are no conflicts to declare.

## Supplementary Material

SC-013-D1SC06857A-s001
