# Supplementary material for: Pressing matter: why are ionic liquids so viscous?
Source: Chem Sci. 2022 Feb 8;13(9):2735–43. doi: 10.1039/d1sc06857a (PMC8890108; doi:10.1039/d1sc06857a)
Supplement: SC-013-D1SC06857A-s001 [file SC-013-D1SC06857A-s001.pdf]

Electronic Supplementary Material (ESI) for Chemical Science.
This journal is © The Royal Society of Chemistry 2022

Supporting information to

Pressing Matter: Why are Ionic Liquids so Viscous?

Frederik Philippi[a] Daniel Rauber[b] Kira Lieberkind Eliasen[c]
Nathalie Bouscharain[d] Kristine Niss[c] Christopher W. M. Kay[b,e]
Tom Welton[a]

January 19, 2022

[a] Department of Chemistry, Molecular Sciences Research Hub, Imperial College London, White City Campus, London W12 0BZ, United Kingdom.

[b] Department of Chemistry, Saarland University, Campus B2.2, Saarbrücken, Germany.

[c] "Glass and Time", IMFUFA, Department of Science and Environment, Roskilde University, P.O. Box 260, DK-4000 Roskilde, Denmark

[d] Univ Lyon, INSA Lyon, CNRS, LaMCoS, UMR5259, 69621 Villeurbanne, France.

[e] London Centre for Nanotechnology, University College London, 17-19 Gordon Street, London WC1H 0AH, UK

Contents

1 Isodensity Conditions in the Literature **3**

1.1 Experimental 3

1.2 MD simulations 4

2 Selection of the Molecular Mimic **5**

2.1 Exploratory Experiments 5

2.2 Volume Calculations 7

3 P-V-T Measurements **7**

3.1 Ambient Pressure 7

3.2 High Pressure 10

4 Viscosity Measurements **14**

4.1 Ambient Pressure 14

4.2 High Pressure 18

5 Syntheses **20**

1 Isodensity Conditions in the Literature

1.1 Experimental

The pair of density and viscosity measurements in Morrison and Lind[1] at the lowest temperature was reported for $[NBu_4][BBu_4]$ at 114.4°C, with a viscosity of 18.68 mPa s and a density of 0.7914 g cm^{-3}. Pressure dependent viscosity data for CBu_4 at 112.37°C can then be used to interpolate linearly between the viscosity values for 0.77128 g cm^{-3} and 0.79747 g cm^{-3}, which gives a viscosity estimate of roughly 2.5 mPa s. For reference, the viscosity of the molecular mimic at ambient pressure at this temperature is less than 0.9220 mPa s.

This heuristic approach can be repeated for a higher temperature to show that the relative differences become smaller. At 162.93°C, the ionic liquid has a viscosity of 5.71 mPa s and at 162.91°C the ionic liquid has a density of 0.7661 g cm^{-3}. Correspondingly, the viscosity of CBu_4 at 164.12°C is 1.13 mPa s under isodensity conditions and about 0.48 mPa s at 160°C, hence the viscosity ratios are somewhere around 2 to 3 for the isodense molecular mimic compared to the molecular mimic under ambient conditions, and 5 for the ionic liquid to the isodense molecular mimic.

The concept can be further expanded using density scaling, see also the pertinent literature for ionic liquids.[2,3] Briefly, isotherms as different pressures can be brought to coincide when scaled appropriately, thus it is possible to compare points with the same ρ^γ/T rather than the stricter choice of isodensity conditions in our present work. Assuming one wishes to investigate the behaviour of a molecular mimic at isodensity conditions (with the density ρ_{IL} and temperature T_{IL} of the corresponding ionic liquid), it might not be feasible to attain this exact combination of density and temperature experimentally, for example due to phase transitions. However, the density ρ and temperature T of the molecular mimic might be chosen so that Equation 1 is fulfilled.

$$\frac{\rho^{\gamma_{MM}}}{T} = \frac{(\rho_{IL})^{\gamma_{MM}}}{T_{IL}} \tag{1}$$

Here, γ is a parameter specific to the molecular mimic. To give an example, we plot the viscosity data from Morrison and Lind as a function of ρ^γ/T, Figure 1. Here, we used $\gamma_{IL} = 2.45881$ for $[NBu_4][BBu_4]$ and $\gamma_{MM} = 3.58777$ for CBu_4. In Figure 1, room temperature conditions would correspond to approximately $\rho^\gamma/T \approx 0.002$.

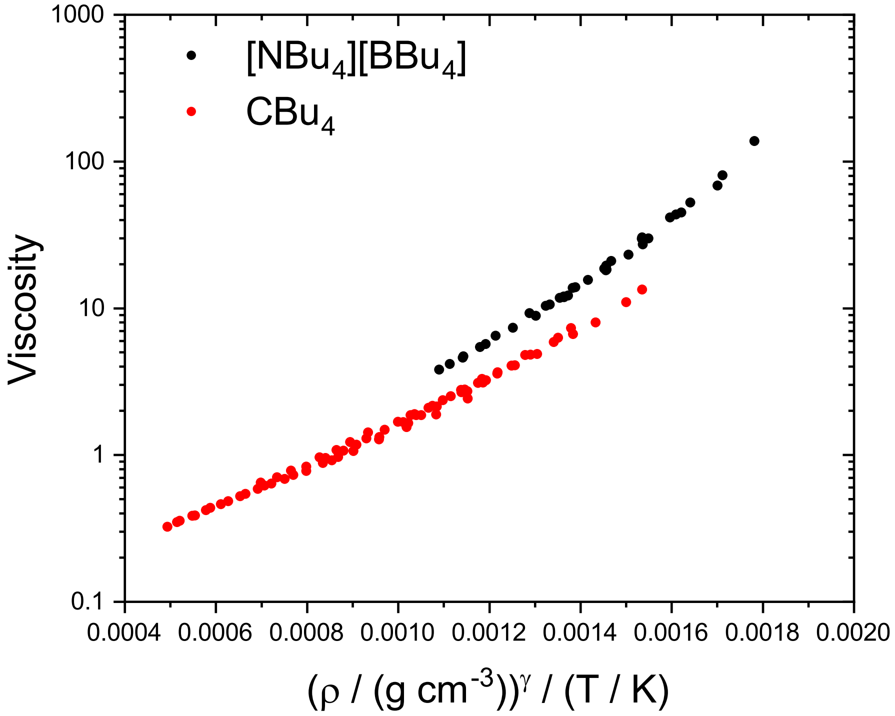

Figure 1: Viscosity values from Morrison and Lind,[1] different isotherms are brought to coincide *via* density scaling.

1.2 MD simulations

Park *et al.* studied three coarse grained model systems: 1) an analogue of an ionic liquid with symmetric charge distribution (SCM), 2) an analogue of an ionic liquid with asymmetric charge distribution (ACM), 3) a molecular mimic by removing all electrostatic interactions (UCM).[4] The diffusion coefficients they obtained from the simulation are shown in Figure 2 as a function of temperature. From the diffusion coefficients, the viscosity ratios can be estimated via the Stokes-Einstein relation, Equation 2.

$$D \propto \frac{1}{\eta} \tag{2}$$

Here, the available data points at the lowest and highest temperature are be compared. The diffusion reported by Park *et al.* increases from 0.0833 nm^2/s at 370 K for the SCM to 1.559 nm^2/s at 375 K for the UCM, and from 0.00202 nm^2/s at 250 K to 0.744 nm^2/s at 250 K (obtained *via* linear interpolation between 225 K and 275 K).

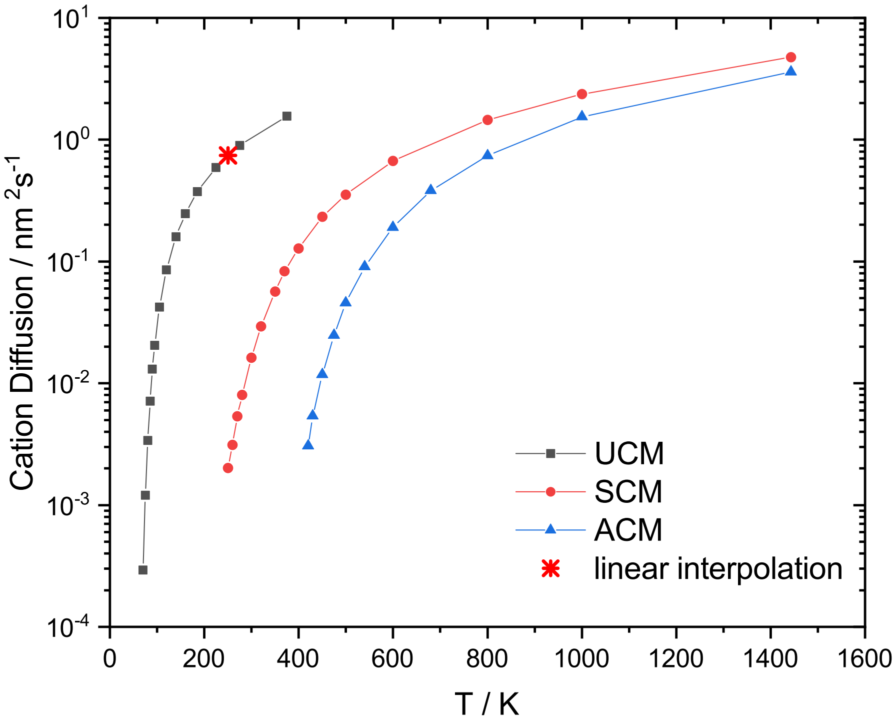

Figure 2: Diffusion coefficients from Part *et al.*[4] UCM = uncharged model, SCM = symmetrically charged model, ACM = asymmetrically charged model.

2 Selection of the Molecular Mimic

2.1 Exploratory Experiments

Here we outline the preliminary experiments which led to the selection of our molecular mimic in the main manuscript. The experiments were conducted on a small scale ($\approx$ 1 cm^3), mixing equimolar amounts of the two components of a potential molecular mimic. Compounds which were hygroscopic and/or reactive were handled in a glovebox.

We first considered pyrroles as molecular mimics of the very common imidazolium cations. Similarly, OTf_2 and CH_2Tf_2 would be suitable molecular mimics of the commonly used $[NTf_2]^-$ anion. However, *N*-butylpyrrole and OTf_2 reacted instantly; turning first green, then brown, and finally forming two layers. Similarly, a solution of *N*-butylpyrrole added to a solution of OTf_2 in $[C_4C_1Im][NTf_2]$ turned brown within 1 min, and the *N*-butylpyrrole reacted completely by the time it took to record an NMR spectrum. However, OTf_2 in $[C_4C_1Im][NTf_2]$ showed no signs of reaction (visually, and via $^1H/^{19}F$ NMR). Adding methylene ditriflone CH_2Tf_2 to *N*-butylpyrrole, no protonation of the *N*-butylpyrrole seemed to occur, but decomposition was clearly visible in the ^1H NMR (After 20 h in bulk, recorded with DMSO-d6 capillary).

Similarly, *N*-butylpyrrole with HCl 37% separated in two phases. The lower phase turned yellow within minutes, containing HCl as well contamination which was not identified, and further separated into two layers later. The initial upper phase contained mostly neat, non-protonated *N*-butylpyrrole. While being miscible, dimethyl sulphate Me_2SO_4 (as an anion neutral analogue of dimethyl phosphate) and *N*-butylpyrrole reacted with each other, with the colour changing from yellow or purple to green within 20 min. As an alternative to pyrroles, we also considered furanes. To this end, we added OTf_2 to 2,5-dimethylfuran. An immediate violent reaction occurred after the addition of the first drop of the triflic anhydride, forming a black residue within seconds. On a side note, while nitromethane was miscible with both 2,5-dimethylfuran and *N*-butylpyrrole without visible reaction, we avoided the use of nitromethane in the high pressure experiments due to its volatility and potential reactivity.

We furthermore considered benzenes as molecular mimics of pyridinium ionic liquids. However, we wanted to avoid the use of the malodinitrile chosen by Shirota and Castner,[5] since this compound might bias result by introducing additional hydrogen bonding. Toluene and OTf_2, in $[C_4C_1Im][NTf_2]$ as solvent, showed only slight yellowing, but no reaction in $^1H/^{19}F$ NMR apart from the formation of a small amount of HOTf. We thus tried mixtures / molecular mimics involving the readily available butylbenzene. OTf_2 and $C(CN)_4$ were not miscible with (soluble in) butylbenzene. However, acetic acid, nitromethane, dimethyl sulphate and acetyl chloride were indeed miscible with butylbenzene without visible reaction. Here, the issue was the use of acetic acid (introducing hydrogen bonding), acetyl chloride (danger of corrosion of the high pressure equipment), and nitromethane (uncertain reactivity and high volatility).

We thus finally decided to turn to molecular mimics of phosphonium ionic liquids, since both the ionic liquids and the molecular mimics are readily accessible experimentally and in general show good chemical and thermal stability. Methyltripentyl silane Si5551 was miscible with acetyl chloride (which could not be used due to corrosing issues), but was not miscible with $C(CN)_4$, acetic acid, OTf_2, dimethyl sulphate or nitromethane. It appeared reasonable to test the smaller silanes, triethylpentyl silane Si2225 as well as triethyl(3-methoxypropyl) silane Si2223O1. We furthermore decided to move to heavier nitroalkanes which are easier to handle due to their higher boiling points. This also improved the miscibility. For example, Si2225 was miscible with nitropropane at room temperature, but separated into two phases when cooling the mixture to 5°C. In contrast, Si2225 and nitrohexane formed a homogeneous mixture down to −20°C, but solidified when placed in dry ice (−78°C). Similarly, Si2223O1 and

nitromethane were immiscible at room temperature, but Si2223O1 / nitropropane remained as a homogeneous, liquid mixture down to dry ice temperatures (−78°C).

2.2 Volume Calculations

Volumes have been calculated at the RB3LYP-GD3BJ/6-311+G(d,p) level of theory.[6,7] To this end, starting geometries have been fully optimised using the Gaussian software package, revision E.01.[8] No symmetry was applied and all calculations have been performed with SCF convergence tightened to 10^{-10} RMS change in the density matrix, extremely tight convergence criteria, and a pruned integration grid with 99 radial shells and 590 angular points per shell. Force constants have been calculated analytically on the stationary points resulting from the geometry optimisation, confirming the stationary points as minima *via* the absence of imaginary frequencies. The wavefunction of the converged geometry was processed using Multiwfn as described in the literature to obtain the volume enclosed by the 0.001 isosurface, see Table 1.[9–11] The constituent volumes of the molecular mimic deviate only by +5% (silane/phosphonium) and −9% (nitropropane/butyrate) from that of the ionic liquid. The molar volume of the ionic liquid (255 cm^3mol^{-1}) and the molecular mimic (257 cm^3mol^{-1}) are virtually identical. Hence, similar free volume can be expected to be present under isodensity conditions.

Table 1: Volumes enclosed by the 0.001 a.u. isosurface.

System	Constituent	Volume / Å^3	Molar Volume / cm^3mol^{-1}
Molecular Mimic	Si222(3O1)	305.8	184.2
	Nitropropane	121.4	73.1
Ionic Liquid	$[P222(3O1)]^+$	291.0	175.2
	$[C_2H_7COO]^-$	133.2	80.2

3 P-V-T Measurements

3.1 Ambient Pressure

The densities of the ionic liquid (triethyl(3-methoxypropy)phosphonium butyrate) and the molecular mimic (equimolar mixture of triethyl(3-methoxypropyl) silane and nitropropane) were measured as a function of temperature, Figure 3 and Figure 4, respectively. The densities

at room temperature and ambient pressure were obtained from the linear fit. We also measured the density of triethylpentylphosphonium valerinate, however we did not use the corresponding molecular mimic for technical reasons. All experimental density values are given in Table 2. The uncertainty of the density values obtained with our setup is approximately 0.1% of the absolute value.[12]

Table 2: Density measurements under ambient pressure. MM = molecular mimic = equimolar mixture of triethyl(3-methoxypropyl) silane and nitropropane, IL = ionic liquid = (triethyl(3-methoxypropy)phosphonium butyrate. Density values are given in g cm^{-3}.

θ / °C	ρ(MM)	ρ(IL)	ρ([P2225][pentanoate])
20	0.878	—	—
25	0.874	1.032	0.962
30	0.870	—	—
35	0.865	1.025	0.956
40	0.861	—	—
45	0.856	1.019	0.949
50	0.852	—	—
55	0.847	1.013	0.943
60	0.842	—	—
65	0.838	1.006	0.936
70	0.833	—	—
75	—	0.999	0.930
85	—	0.992	0.924
95	—	0.986	0.917

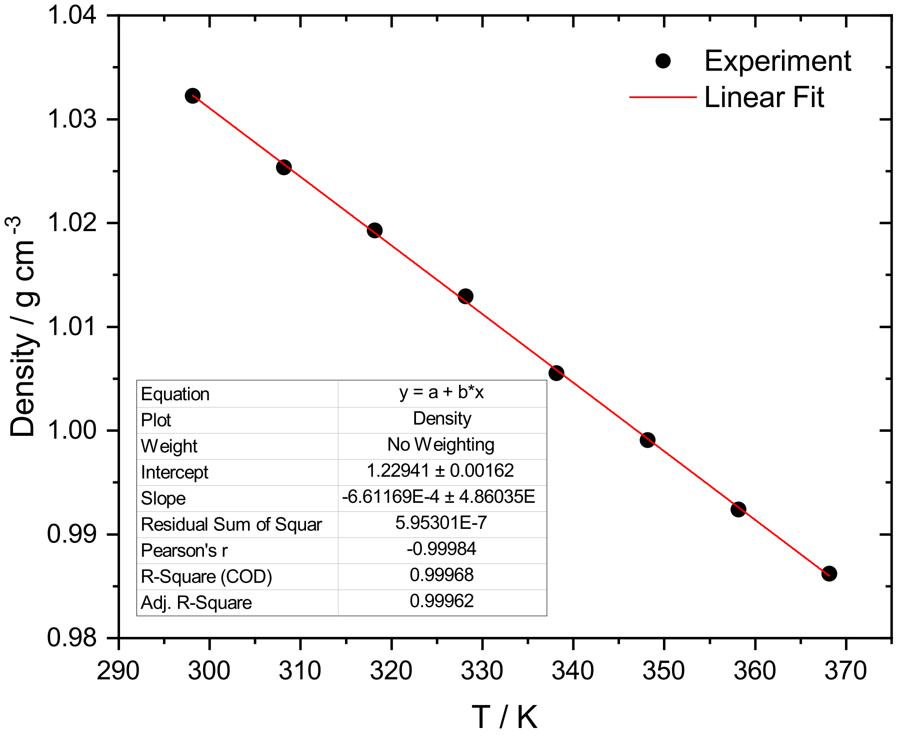

Figure 3: Density of the ionic liquid as a function of temperature at ambient pressure.

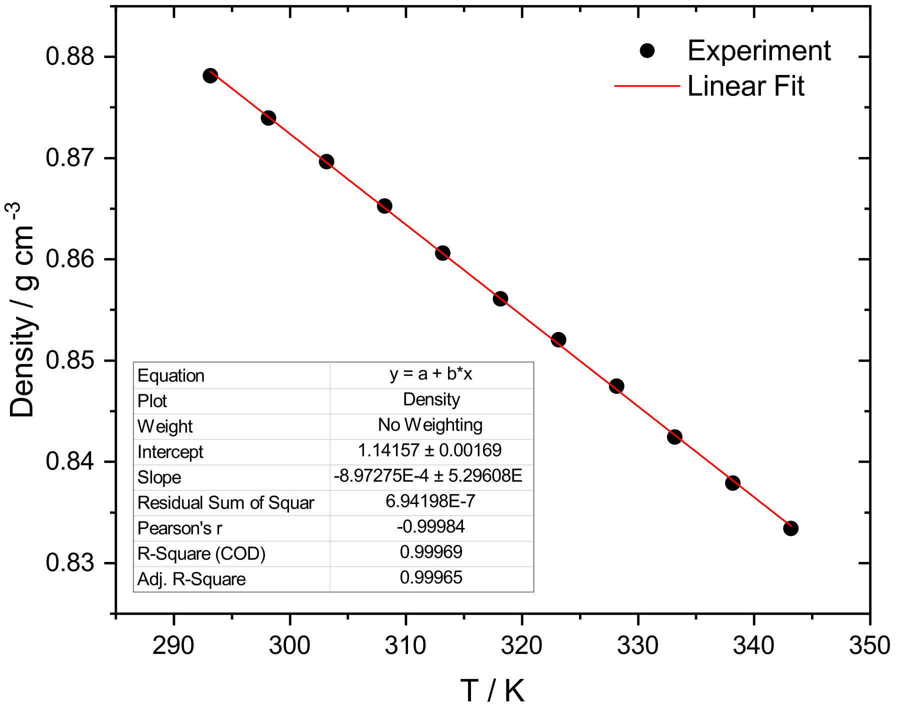

Figure 4: Density of the molecular mimic as a function of temperature at ambient pressure.

3.2 High Pressure

The density ρ as a function of pressure P was obtained from the displacement Δh of the piston pressurising the sample of mass m placed in a cylindrical container with a bore radius r of 10 mm, Equation 3. The height of the sample in the cylinder bore was calculated from the room temperature density under ambient pressure ρ_0, Equation 4

$$\rho = \frac{m}{(h - \Delta h)\pi r^2} \tag{3}$$

$$h = \frac{m}{\rho_0 \pi r^2} \tag{4}$$

Two independently synthesised samples of the molecular mimic were measured, and for each of those samples, two repeats were performed. The first sample had a mass of 11.335 g and was measured up to 300 MPa, the data are presented in Table 3. The second sample had a mass of 11.3150 g and was measured up to 350 MPa, the data are presented in Table 4.

Table 3: Density measurements on the first sample.

First measurement				Second Measurement			
T / K	P / bar	Δh / mm	ρ / g cm^{-3}	T / K	P / bar	Δh / mm	ρ / g cm^{-3}
297.99	18	0.258	0.8795	298.01	3	0.226	0.8788
298.00	117	0.566	0.8862	297.99	123	0.582	0.8865
298.03	220	0.846	0.8923	298.00	214	0.844	0.8922
297.99	320	1.098	0.8979	298.01	319	1.096	0.8978
297.98	419	1.328	0.9031	298.01	420	1.334	0.9032
297.99	518	1.552	0.9081	298.00	519	1.554	0.9082
298.00	618	1.768	0.9131	298.01	622	1.776	0.9133
298.00	717	1.970	0.9178	298.01	720	1.974	0.9179
298.02	815	2.168	0.9224	298.00	822	2.166	0.9224
298.00	915	2.360	0.9270	297.99	918	2.348	0.9267
298.01	1016	2.568	0.9320	298.00	1020	2.528	0.9310
298.03	1116	2.742	0.9362	298.04	1120	2.700	0.9352
298.01	1216	2.916	0.9404	297.99	1219	2.862	0.9391
298.01	1312	3.086	0.9446	297.99	1320	3.026	0.9431
298.01	1414	3.242	0.9485	297.98	1420	3.182	0.9470
298.00	1514	3.392	0.9522	298.00	1519	3.326	0.9506
298.01	1613	3.556	0.9564	297.99	1619	3.472	0.9543
297.99	1714	3.694	0.9599	297.97	1716	3.606	0.9577
298.02	1814	3.834	0.9635	297.99	1821	3.748	0.9613
297.98	1913	3.970	0.9670	298.01	1916	3.874	0.9645
298.00	2012	4.096	0.9703	298.01	2018	4.002	0.9678
298.02	2112	4.226	0.9737	297.99	2117	4.128	0.9711
298.00	2212	4.352	0.9770	297.99	2216	4.246	0.9742
298.00	2316	4.480	0.9804	298.00	2315	4.364	0.9773
297.99	2411	4.584	0.9832	298.00	2416	4.482	0.9805
298.02	2512	4.690	0.9860	298.01	2513	4.594	0.9834
298.01	2613	4.794	0.9888	298.01	2617	4.710	0.9866
298.01	2713	4.898	0.9917	298.00	2715	4.814	0.9894
298.03	2811	4.994	0.9943	297.98	2816	4.914	0.9921
297.99	2914	5.094	0.9970	298.00	2913	5.016	0.9949
297.99	3018	5.188	0.9996	298.00	3012	5.116	0.9976
				298.00	3112	5.214	1.0004
				298.00	3195	5.292	1.0025
				298.00	3295	5.384	1.0051

Table 4: Density measurements on the second sample.

First measurement				Second Measurement			
T / K	P / bar	Δh / mm	ρ / g cm^{-3}	T / K	P / bar	Δh / mm	ρ / g cm^{-3}
298.02	3	0.096	0.8756	298.00	3	0.076	0.8756
298.00	120	0.442	0.8831	298.00	117	0.424	0.8831
297.99	219	0.732	0.8895	298.00	216	0.718	0.8895
298.00	318	0.982	0.8951	298.00	317	0.970	0.8951
298.00	419	1.218	0.9003	298.01	418	1.204	0.9003
298.00	518	1.438	0.9053	298.00	518	1.426	0.9053
297.98	617	1.648	0.9102	297.99	619	1.640	0.9102
297.99	718	1.856	0.9148	298.00	716	1.838	0.9148
298.01	817	2.048	0.9194	298.00	818	2.034	0.9194
297.99	917	2.236	0.9238	298.00	917	2.222	0.9238
298.00	1014	2.414	0.9281	298.00	1017	2.402	0.9281
298.01	1117	2.596	0.9323	298.00	1114	2.576	0.9323
298.00	1216	2.762	0.9365	298.01	1217	2.748	0.9365
298.01	1315	2.920	0.9403	298.00	1315	2.906	0.9403
297.99	1417	3.078	0.9442	298.03	1417	3.062	0.9442
297.99	1516	3.226	0.9478	298.01	1516	3.210	0.9478
298.01	1616	3.372	0.9514	298.00	1616	3.354	0.9514
298.01	1714	3.510	0.9549	298.00	1713	3.492	0.9549
298.00	1816	3.650	0.9584	297.99	1816	3.630	0.9584
297.99	1914	3.780	0.9619	297.99	1918	3.766	0.9619
298.00	2015	3.910	0.9651	298.00	2016	3.890	0.9651
298.01	2115	4.034	0.9683	298.00	2116	4.012	0.9683
298.01	2217	4.158	0.9715	297.99	2216	4.134	0.9715
297.99	2317	4.274	0.9745	298.01	2314	4.248	0.9745
298.01	2416	4.386	0.9774	298.00	2409	4.360	0.9774
298.00	2513	4.494	0.9806	298.00	2517	4.478	0.9806
297.99	2613	4.602	0.9834	298.00	2618	4.586	0.9834
298.03	2712	4.708	0.9862	298.01	2713	4.688	0.9862
298.00	2802	4.804	0.9890	298.00	2814	4.792	0.9890
298.00	2890	4.894	0.9917	298.01	2913	4.892	0.9917
298.00	3007	5.008	0.9943	298.00	3008	4.986	0.9943
298.00	3100	5.100	0.9972	297.99	3094	5.090	0.9972
298.00	3201	5.200	0.9996	298.00	3189	5.176	0.9996
298.01	3299	5.292	1.0026	298.00	3310	5.284	1.0026
298.01	3399	5.378	1.0049	298.02	3400	5.368	1.0049
297.99	3478	5.462	1.0063	298.01	3491	5.418	1.0063

The experimental data points for both samples were fitted with the three-parameter (isothermal) Tait equation (5).

$$\rho/(\mathrm{g\ cm^{-3}}) = \frac{1}{A(1 - B\ln(1 + \frac{P/bar}{C}))} \tag{5}$$

Here, A, B and C are parameters. The fit results for the two samples are given in Table 5.

Table 5: Tait equation fit parameters, standard errors from the fit given in brackets.

	Sample 1	Sample 2
A	1.1383(6)	1.1416(1)
B	0.1060(24)	0.1068(4)
C	1427(57)	1466(11)
R^2	0.99928	0.99997

The experimental density of the first sample is shown in Figure 5. Here, the isodensity conditions occurred at 438 MPa. The reproducibility was better for the second sample, for which isodensity conditions occurred at 458 MPa. In addition, the second sample was measured to higher pressures; hence, we only used the P-V-T data for the second sample.

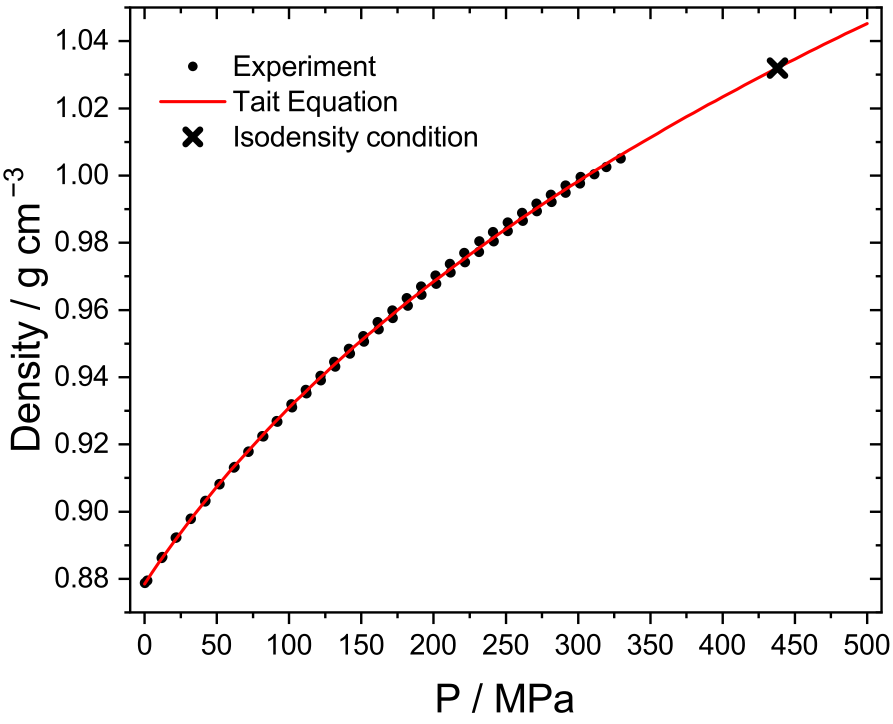

Figure 5: Density of the first sample of molecular mimic at elevated pressure, both measurements.

4 Viscosity Measurements

4.1 Ambient Pressure

The viscosities of the ionic liquid (triethyl(3-methoxypropy)phosphonium butyrate) and the molecular mimic (equimolar mixture of triethyl(3-methoxypropyl) silane and nitropropane) were measured as a function of temperature, Figure 6 and Figure 7, respectively. The experimental data are given in Table 6. The uncertainty in the viscosity measurements is approximately 1%, as determined from repeated measurements of a viscosity standard (129 mPas at 25°C).

The experimental viscosity of the molecular mimic at room temperature with the cone-plate setup was 0.86 mPa s, with the sinker setup the viscosity was approximately 1 mPa s. Hence, there is good agreement between the methods. Even when the viscosity from the cone-plate setup setup is used to calculate the viscosity ratios in the main manuscript, the viscosity ratio between isodense molecular mimic and ambient pressure molecular mimic increases from ≈ 14 to ≈ 16, thus endorsing the conclusions drawn. However, the cone-plate setup was optimised for viscous (ionic liquids), hence we repeated the measurement of the viscosity of the molecular mimic with a coaxial setup at room temperature. The results are shown in Table 7. Excluding the first two points, the average viscosity of the molecular mimic at room temperature is 0.99 mPa s, with a average deviation from the mean of 0.04 mPa s.

The samples showed Newtonian behaviour, *i.e.* no significant variation of the viscosity with the shear rate was observed.

Table 6: Viscosity measurements under ambient pressure, cone-plate setup. MM = Molecular mimic = equimolar mixture of triethyl(3-methoxypropyl) silane and nitropropane, IL = ionic liquid = (triethyl(3-methoxypropy)phosphonium butyrate. Viscosity values are given in mPa s.

θ / °C	T / K	η(MM)	η(IL)	θ / °C	T / K	η(MM)	η(IL)
25	298.15	0.85	217.2	70	343.15	0.48	27.2
30	303.15	0.77	161.8	75	348.15	0.44	23.0
35	308.15	0.76	123.3	80	353.15	0.41	19.6
40	313.15	0.70	95.1	85	358.15	0.39	16.9
45	318.15	0.64	74.7	90	363.15	0.36	14.7
50	323.15	0.61	59.6	95	368.15	0.35	12.8
55	328.15	0.57	48.1	100	373.15	0.33	11.3
60	333.15	0.54	39.3	105	378.15	0.30	10.0
65	338.15	0.51	32.6				

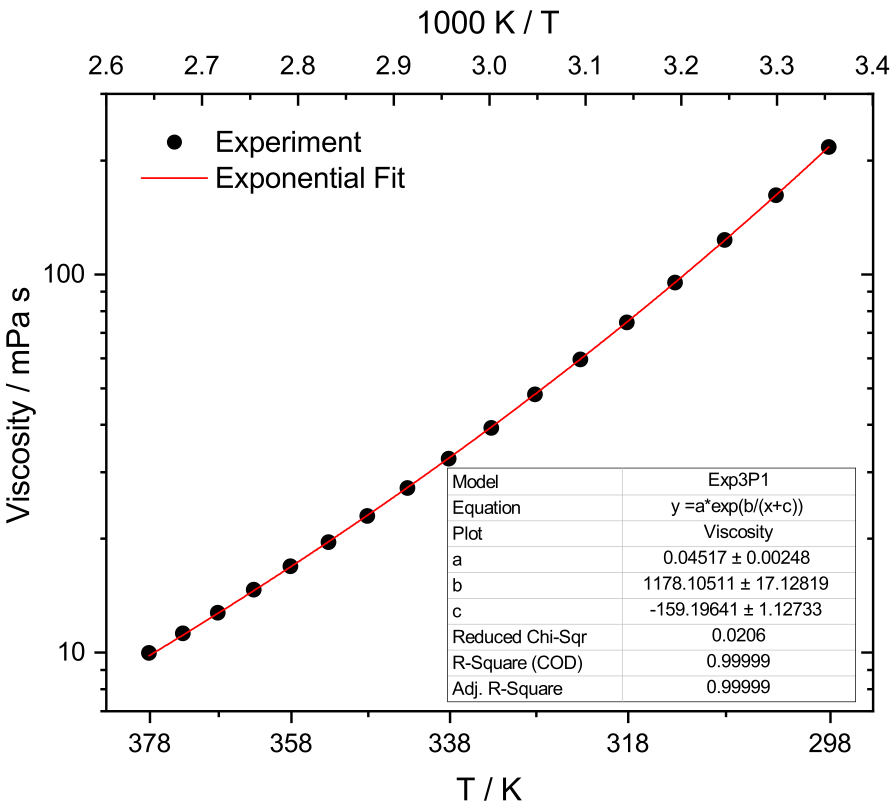

Figure 6: Viscosity of the ionic liquid as a function of temperature at ambient pressure.

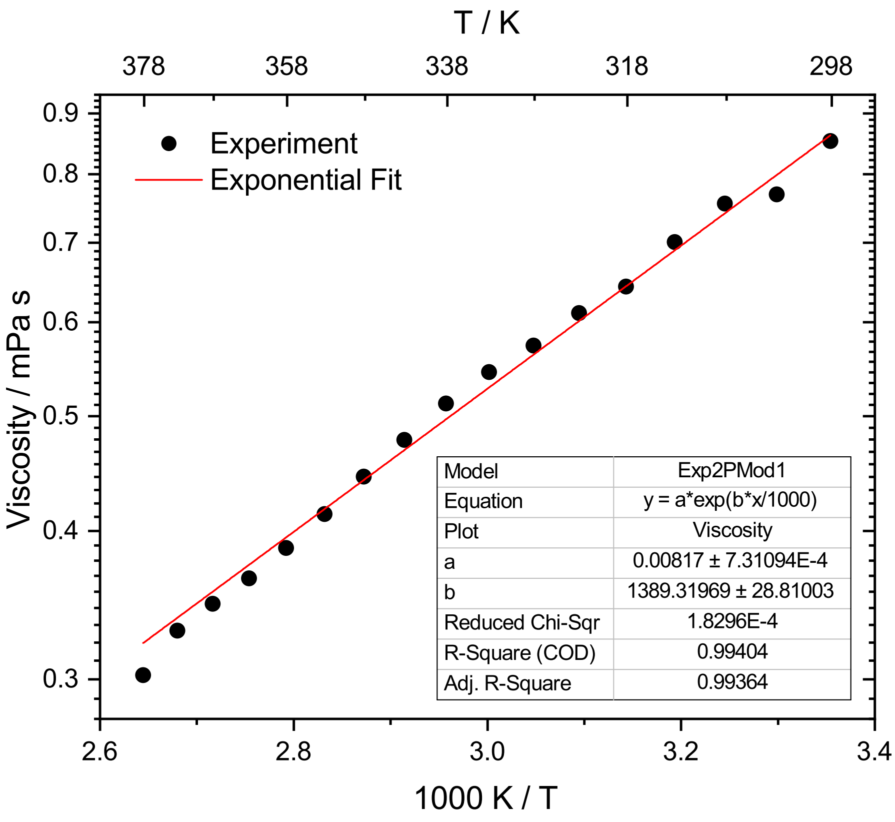

Figure 7: Viscosity of the molecular mimic as a function of temperature at ambient pressure.

Table 7: Viscosity measurements for the molecular mimic under ambient pressure at ambient temperature, coaxial setup.

shear rate / s^{-1}	shear stress / mPa	Torque / μN m	Viscosity
1.00	0.86392	0.0458	0.86
1.19	0.87639	0.0464	0.74
1.41	1.2658	0.0671	0.90
1.68	1.6062	0.0851	0.96
2.00	2.1360	0.113	1.07
2.37	2.4240	0.128	1.02
2.82	2.5244	0.134	0.90
3.35	3.1516	0.167	0.94
3.98	4.0265	0.213	1.01
4.73	4.3187	0.229	0.91
5.62	5.5953	0.296	1.00
6.68	6.2365	0.330	0.93
7.94	7.5465	0.400	0.95
9.44	9.1875	0.487	0.97
11.20	10.941	0.580	0.98
13.30	12.719	0.674	0.95
15.80	15.371	0.814	0.97
18.80	18.199	0.964	0.97
22.40	22.100	1.17	0.99
26.60	26.701	1.41	1.00
31.60	32.520	1.72	1.03
37.60	38.518	2.04	1.02
44.70	45.731	2.42	1.02
53.10	56.127	2.97	1.06
63.10	66.093	3.50	1.05
75.00	79.223	4.20	1.06
89.10	97.537	5.17	1.09

4.2 High Pressure

The viscosity of the molecular mimic was measured up to 500 MPa at room temperature using a falling body viscometer. The viscosity at a given pressure is obtained from the calibration factor C of the falling body (=sinker), the falling time t, the density ρ of the liquid and the density ρ_s of the falling body, Equation 6.

$$\eta = C \cdot t \cdot \frac{\rho_s - \rho}{\rho_s} \tag{6}$$

Due to the low viscosity of the molecular mimic, one falling body with a density of $\rho_s = 7.75$ g cm^{-3} and a calibration factor $C = 7.3226$ was sufficient to cover the whole pressure range. The resulting data are presented in Table 8 and Figure 8. The viscosity under isodensity conditions was obtained *via* interpolation. To this end, the viscosity was fitted as a function of density with $\eta = a \cdot b^{(\rho^c)}$, Figure 9.

Table 8: Falling times and resulting viscosity values as a function of pressure. The standard deviations of the falling times t across 4-6 repeats is given in brackets. Using three times this standard deviation with Gaussian error propagation gives the uncertainty estimate in the viscosity η (in brackets).

P / bar	t / s	η / mPa s
1	0.154(1)	1.00(2)
250	0.191(1)	1.24(1)
500	0.234(4)	1.51(8)
1000	0.333(0)	2.15(1)
1500	0.456(1)	2.93(2)
2000	0.603(2)	3.87(3)
2500	0.785(3)	5.02(6)
3000	1.008(4)	6.43(7)
3500	1.280(2)	8.16(4)
4000	1.631(14)	10.37(27)
4500	2.038(14)	12.94(27)
5000	2.541(4)	16.11(8)

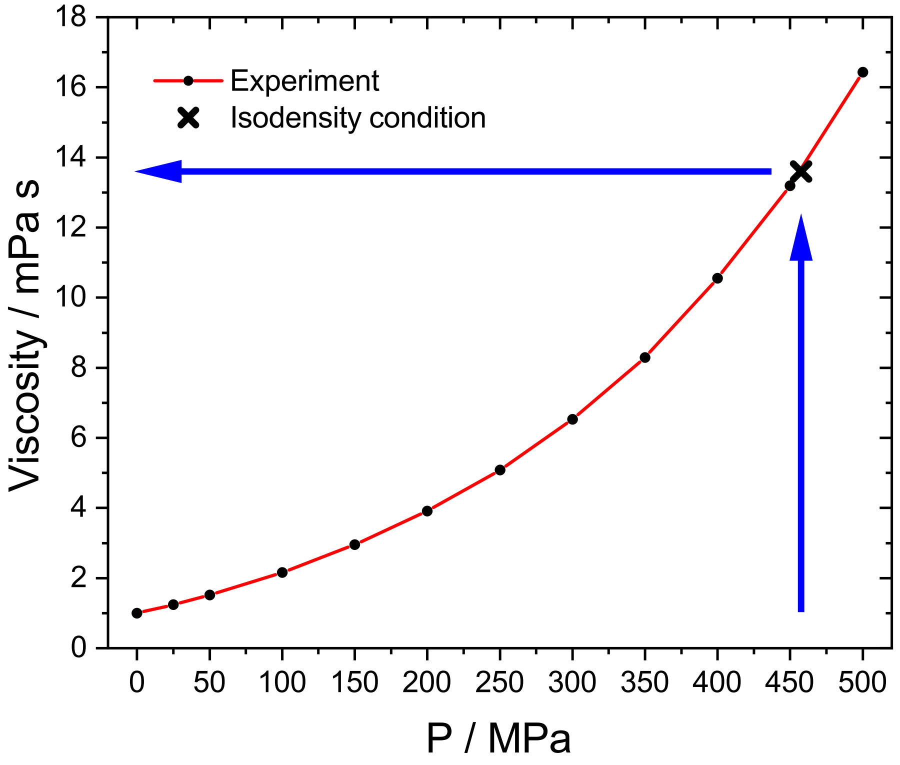

Figure 8: Viscosity of the molecular mimic as a function of pressure at room temperature.

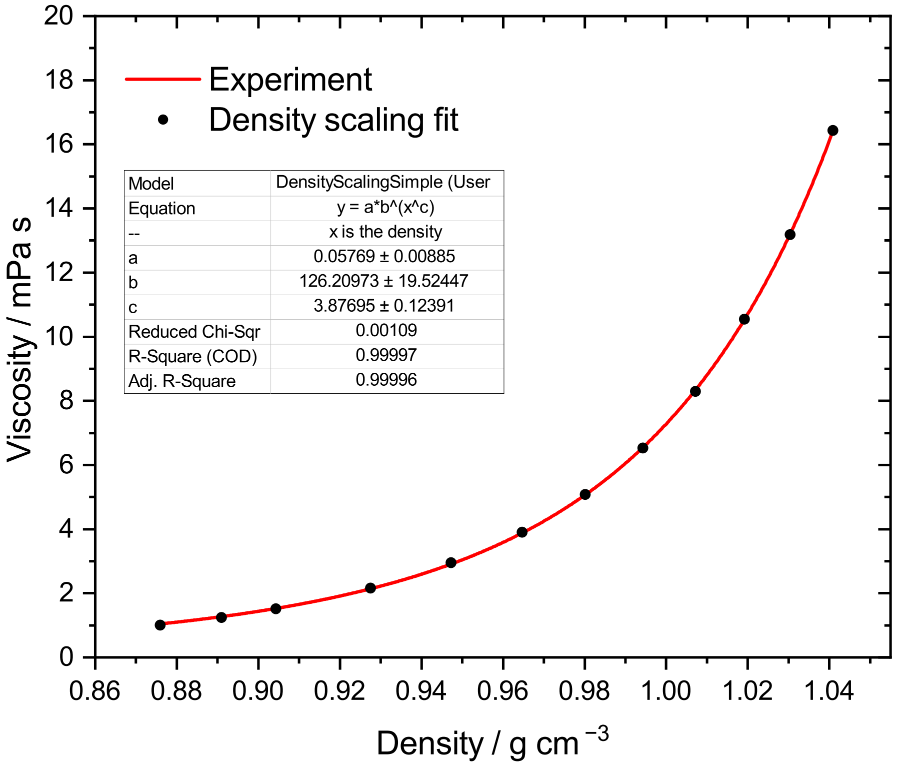

Figure 9: Viscosity of the molecular mimic as a function of density at room temperature. The red line connecting the experimental data points is a guide for the eye.

5 Syntheses

***N*-Butylpyrrole** (Based on [13], Schlenk conditions until workup) A 100 mL two necked round bottom flask was charged with 10.3 g potassium hydroxide 85% (156 mmol / 1.96 eq) and 40 mL dimethyl sulfoxide. After stirring for 10 min, the suspension was cooled to 0°C, and 5.32 g pyrrole (79.3 mmol / 1.00 eq) were added. The resulting mixture was stirred at ambient temperature for 30 min. Then, 10 mL 1-bromobutane (12.6 g / 92.0 mmol / 1.16 eq) were added slowly over 45 min with the reaction flask being immersed in a water bath as heat sink. After addition was complete, the reaction mixture was stirred for 16 h. Then, the contents of the flask were poured into 500 mL water and subsequently extracted with 200 mL, 100 mL, and 50 mL CH_2Cl_2. The combined organic phases were washed thrice with 100 mL water each, then with 100 mL brine. The organic phase was then dried with Na_2SO_4 overnight, filtered, and the CH_2Cl_2 removed at 30°C under reduced pressure using a rotary evaporator. The resulting liquid was distilled from potassium hydroxide in vacuum (bath temperature 30°C), giving 7.81 g the title compound (63.4 mmol / 80%). ^{1}H NMR (DMSO−d$_6$, 400 MHz, δ in ppm): 6.73 (t, $^3J_{H/H}$ = 2.1 Hz, 2H, N−**CH**=CH), 6.22 (t, $^3J_{H/H}$ = 2.0 Hz, 2H, N−CH=**CH**), 3.95 (t, $^3J_{H/H}$ = 7.2 Hz, 2H, N−**CH$_2$**), 1.83 (p, $^3J_{H/H}$ = 7.3 Hz, 2H, N−CH$_2$−**CH$_2$**), 1.40 (h, $^3J_{H/H}$ = 7.4 Hz, 2H, N−(CH$_2$)$_2$−**CH$_2$**−CH$_3$), 3.02 (t, $^3J_{H/H}$ = 7.4 Hz, 2H, N−(CH$_2$)$_3$−**CH$_3$**); ^{13}C{^{1}H} NMR (DMSO−d$_6$, 101 MHz, δ in ppm): 120.50 (s, N−**CH**=CH), 107.82 (s, N−CH=**CH**), 49.38 (s, N−**CH$_2$**), 33.70 (s, N−CH$_2$−**CH$_2$**), 20.01 (s, N−(CH$_2$)$_2$−**CH$_2$**−CH$_3$), 13.71 (s, N−(CH$_2$)$_3$−**CH$_3$**).

Molecular Mimic The molecular mimic used in the main manuscript was prepared by mixing 14.451 g Si222(3O1) (76.7 mmol, 1.00 eq) with 6.833 g 1-nitropropane (76.7 mmol, 1.00 eq) inside a glovebox.

[P222(3O1)][Br]: 6.26 mL of triethyl phosphine (5.02 g / 42.5 mmol / 1.00 eq) were dissolved in 200 mL of dry, degassed acetonitrile under argon and 7.12 mL of 1-bromo-3-methoxypropane (9.76 g / 63.8 mmol / 1.50 eq) were added. The resulting homogeneous solution was stirred for 5 days at ambient temperature. The solvent and excess of bromo-ether were removed by rotary evaporation and the residue dried in high vacuum to obtain 11.3 g triethyl(3-methoxypropyl) phosphonium bromide (41.7 mmol/ 98% yield) as white solid. ^{1}H NMR ($CDCl_3$, 400 MHz, δ in ppm): 3.38 (t, $^3J_{H/H}$ = 5.6 Hz, 2H, O−**CH$_2$**), 3.22 (s, 3H, O−CH$_3$), 2.48-2.36 (m, 8H, P−CH$_2$), 1.81 (ttd, $^3J_{H/H}$ = 15.8 Hz, $^3J_{H/H}$ = 15.8 Hz, $^3J_{P/H}$ = 5.6 Hz, 2H, **CH$_2$**−CH$_2$), 1.20 (dt, $^3J_{H/P}$ = 18.1 Hz, $^3J_{H/H}$ = 7.7 Hz, 9H, CH$_2$−**CH$_3$**); ^{13}C{^{1}H}

NMR ($CDCl_3$, 101 MHz, δ in ppm): 71.12 (d, $^4J_{H/P}$ = 13.0 Hz, $\mathbf{CH_2}$−O), 58.61 (s, O−CH_3), 22.15 (d, $^3J_{H/P}$ = 4.4 Hz, CH_2−$\mathbf{CH_2}$−CH_2), 15.26 (d, $^2J_{H/P}$ = 49.1 Hz, P−$\mathbf{CH_2}$−CH_2), 12.39 (d, $^2J_{H/P}$ = 49.0 Hz, P−$\mathbf{CH_2}$−CH_3), 5.96 (d, $^3J_{H/P}$ = 5.5 Hz, CH_2−$\mathbf{CH_3}$). P−$\mathbf{CH_3}$); $^{31}P\{^1H\}$ NMR ($CDCl_3$, 162 MHz, δ in ppm): 38.96 (s). Elemental analysis: calculated for $C_{10}H_{24}BrOP$: C, 44.29; H, 8.92; N, 0.00. Found C, 44.27; H, 8.95; N, 0.00.

[P222(3O1)][$\mathbf{O_2C(CH_2)_3H}$]: 8.22 g of [P222(3O1)][Br] (30.3 mmol / 1.00 eq) were dissolved in deionized water and the obtained homogeneous solution passed through an Amberlyst A-27 anion exchange resign. The obtained hydroxide solution of the phosphonium cation was immediately neutralized with a dilute aqueous solution of butyric acid (monitored using a digital pH meter). The neutralized solution was dried using an rotary evaporator and the residue dried on a Schlenk line for two days with stirring, final water content was 1360 ppm ($\approx$ 0.1%) as determined by Karl-Fischer titration. The product was obtained as a colorless liquid (8.35 g/ 30.0 mmol/ 99% yield). The DSC trace is shown in Figure 10. ^1H NMR ($CDCl_3$, 400 MHz, δ in ppm): 3.37 (t, $^3J_{H/H}$ = 6.1 Hz, 2H, O−$\mathbf{CH_2}$), 3.23 (s, 3H, O−CH_3), 2.46-2.34 (m, 8H, P−CH_2), 2.03 (t, $^3J_{H/H}$ = 7.4 Hz, 2H, O_2C−CH_2), 1.78 (ttd, $^3J_{H/H}$ = 15.7 Hz, $^3J_{H/H}$ = 9.9 Hz, $^3J_{P/H}$ = 5.6 Hz, 2H, P−CH_2−$\mathbf{CH_2}$), 1.52 (tq, $^3J_{H/H}$ = 7.5 Hz, $^3J_{H/H}$ = 7.5 Hz, 2H, O_2C−CH_2−$\mathbf{CH_2}$), 1.17 (dt, $^3J_{H/P}$ = 18.0 Hz, $^3J_{H/H}$ = 7.7 Hz, 9H, P−CH_2−$\mathbf{CH_3}$), 0.81 (t, $^3J_{H/H}$ = 7.4 Hz, 3H, O_2C−$(CH_2)_2$−$\mathbf{CH_3}$); $^{13}C\{^1H\}$ NMR ($CDCl_3$, 101 MHz, δ in ppm): 179.21 (s, O_2C) 71.32 (d, $^4J_{H/P}$ = 13.2 Hz, $\mathbf{CH_2}$−O), 58.66 (s, O−CH_3), 41.35 (s, O_2C−$\mathbf{CH_2}$), 22.13 (d, $^3J_{H/P}$ = 4.4 Hz, P−CH_2−$\mathbf{CH_2}$), 20.36 (s, O_2C−CH_2−$\mathbf{CH_2}$), 14.96 (d, $^2J_{H/P}$ = 49.1 Hz, P−$\mathbf{CH_2}$−CH_2), 14.51 (s, O_2C−$(CH_2)_2$−$\mathbf{CH_3}$), 11.89 (d, $^2J_{H/P}$ = 49.0 Hz, P−$\mathbf{CH_2}$−CH_3), 5.85 (d, $^3J_{H/P}$ = 5.6 Hz, P−CH_2−$\mathbf{CH_3}$); $^{31}P\{^1H\}$ NMR ($CDCl_3$, 162 MHz, δ in ppm): 39.10 (s). Elemental analysis: calculated for $C_{14}H_{31}O_3P$: C, 60.41; H, 11.23; N, 0.00. Found C, 55.92; H, 11.50; N, 0.11.

Si5551 (The Grignard reagent was prepared under Schlenk conditions until workup. Based on [14, 15]) 3.62 g Magnesium turnings (149 mmol / 3.57 eq) were activated by dry stirring under vacuum, and 22.5 g bromopentane (149 mmol / 3.57 eq) in 100 mL dry THF were added dropwise over 1.5 h. After addition was complete, the reaction mixture was stirred at 40°C for 2 h, and 6.23 g $MeSiCl_3$ (41.7 mmol / 1.00 eq) were added dropwise over 20 min. The mixture was heated to reflux for 7 h and stirred at ambient temperature overnight. Then, 70 mL half saturated aqueous NH_4Cl solution were added. The phases were separated and the aqueous phase extracted twice with 60 mL and 40 mL pentane. The organic phases were combined and the solvent was removed under reduced pressure on a rotary evaporator. Then, 100 mL

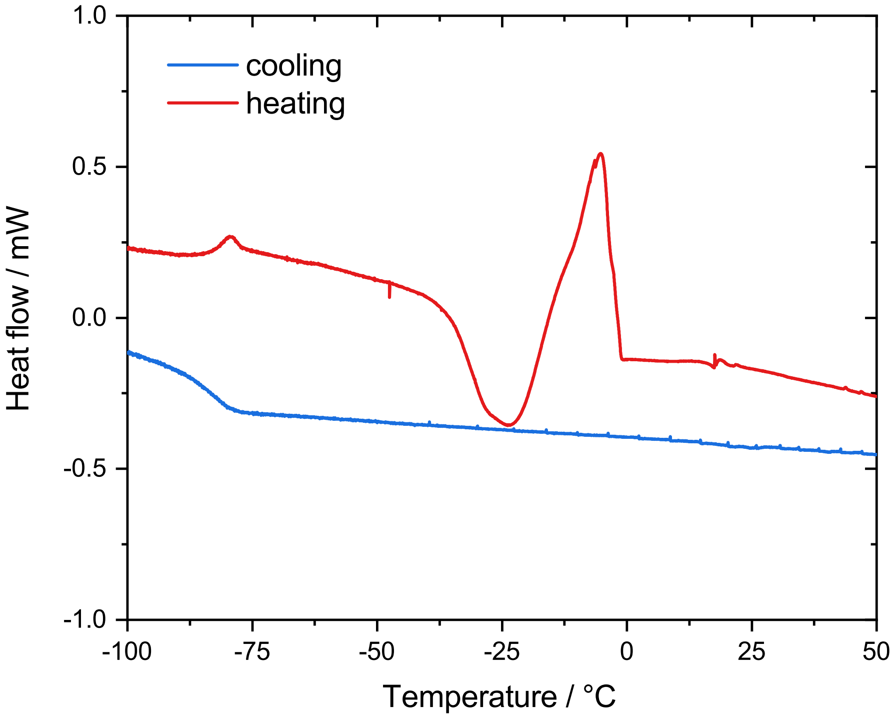

Figure 10: DSC trace of the ionic liquid P2223O1butyrat.

pentane were added to the residue, and the organic phase washed thoroughly with H_2SO_4 50% (v/v) (two times, 20 mL each), 20 mL H_2O, and 20 mL brine. After removal of the solvent under reduced pressure on a rotary evaporator and drying in vacuum, the residue was distilled from P_4O_{10} in high vacuum, giving 8.99 g methyltripentyl silane (35.0 mmol / 84%). ^{1}H NMR ($CDCl_3$, 400 MHz, δ in ppm): 1.39-1.14 (m, 18H, $(\mathbf{CH_2})_3{-}CH_4$), 0.88 (t, $^3J_{H/H}$ = 6.8 Hz, 9H, $CH_2{-}\mathbf{CH_3}$), 0.54-0.37 (m, 6H, Si$-\mathbf{CH_2}{-}CH_2$), -0.08 (s, 3H, Si$-\mathbf{CH_3}$); ^{29}Si NMR ($CDCl_3$, 80 MHz, δ in ppm): 2.84 (s); ^{13}C{^{1}H} NMR ($CDCl_3$, 101 MHz, δ in ppm): 36.22 (s), 23.73 (s), 22.51 (s), 14.20 (s), 13.98 (s), -4.99 (s, Si$-\mathbf{CH_3}$). Elemental analysis: calculated for $C_{16}H_{36}Si$: C, 74.91; H, 14.14; N, 0.00. Found C, 73.33; H, 13.65; N, 0.00.

Ag[C(CN)$_3$] (Based on [16]) The synthesis was conducted in the dark. To 5.42 g Na[C(CN)$_3$] (47.9 mmol / 1.00 eq) dissolved in 65 mL water were added 8.48 g $AgNO_3$ (50.0 mmol / 1.04 eq) dissolved in 60 mL water. The resulting suspension was stirred vigorously for 17 h, with the reaction vessel covered in aluminium foil to exclude light. After that, the reaction mixture was filtered, and the filter cake washed with water followed by ethanol. Drying in high vacuum gave 9.10 g silver tricyanomethanide (46.0 mmol / 96%).

C(CN)$_4$ (Based on [16] and [17], also *cf* [18]) 3.07 g Ag[C(CN)$_3$] (15.5 mmol / 1.00 eq) and 2.37 g cyanogen bromide (22.4 mmol / 1.44 eq) were transferred to a 20 mL pressure tube

(Ace Glass, Vineland, USA) and heated to 100°C for 11 days. The contents of the reaction tube were then kept at a vacuum of 14 mbar at ambient temperature for 3 h to remove any residual cyanogen bromide. Sublimation in high vacuum at 100°C bath temperature using a cold finger cooled with dry ice gave 0.88 g $C(CN)_4$ (7.58 mmol / 49%). Solid state IR spectrum (cm^{-1}): 878 (m), 891 (m), 1028 (m), 1055 (v), 1098 (w), 1126 (m), 2279 (m) (v = very intense, m = medium intense, w = weak). $^{13}C\{^1H\}$ NMR (CD_3CN, 101 MHz, δ in ppm): 103.17 (s, $C(\mathbf{CN})_4$), 22.74 (s, $\mathbf{C}(CN)_4$). Elemental analysis: calculated for $C(CN)_4$: C, 55.01; H, 8.66; N, 16.04. Found C, 53.69; H, 9.14; N, 15.35.

Si2225 (The Grignard reagent was prepared under Schlenk conditions until workup) 3.67 g Magnesium turnings (151 mmol / 1.23 eq) were activated by dry stirring under vacuum, and 19 mL bromopentane (23.0 g / 152 mmol / 1.24 eq) in 100 mL dry THF were added dropwise over 1.5 h at 30°C. After addition was complete, the reaction mixture was stirred at 40°C for 2 h, and 18.5 g Et_3SiCl (123 mmol / 1.00 eq) were added dropwise over 15 min. The mixture was heated to reflux for 24 h and then quenched by slowly adding 80 mL half saturated aqueous NH_4Cl solution, followed by stirring for 2 h. The phases were separated and the aqueous phase extracted with 60 mL and 50 mL pentane. The organic phases were combined and the solvents removed under reduced pressure using a rotary evaporator. The residue was taken up with 100 mL pentane and stirred for 2 h with 20 mL H_2SO_4 50% (v/v). The organic phase was then washed with 20 mL H_2O and 20 mL brine and dried over $MgSO_4$. The solvent was removed under reduced pressure using a rotary evaporator, and the residue distilled from P_4O_{10} in high vacuum, giving 18.9 g triethylpentyl silane (101 mmol / 82%). ^1H NMR ($CDCl_3$, 400 MHz, δ in ppm): 1.37-1.21 (m, 6H, $(\mathbf{CH_2})_3{-}CH_3$), 0.93 (t, $^3J_{H/H}$ = 7.9 Hz, 9H, $Si{-}CH_2{-}\mathbf{CH_3}$), 0.91-0.85 (m, 3H, $CH_2{-}CH_2{-}\mathbf{CH_3}$), 0.54-0.46 (m, 2H, $Si{-}\mathbf{CH_2}{-}CH_2$), 0.50 (q, $^3J_{H/H}$ = 7.9 Hz, 6H, $Si{-}\mathbf{CH_2}{-}CH_3$); ^{29}Si NMR ($CDCl_3$, 80 MHz, δ in ppm): 6.67 (s); $^{13}C\{^1H\}$ NMR ($CDCl_3$, 101 MHz, δ in ppm): 36.36 (s), 23.67 (s), 22.52 (s), 14.20 (s, $CH_2{-}CH_2{-}\mathbf{CH_3}$), 11.44 (s, $Si{-}\mathbf{CH_2}{-}CH_2$), 7.63 (s, $Si{-}CH_2{-}\mathbf{CH_3}$), 3.52 (s, $Si{-}\mathbf{CH_2}{-}CH_3$).

Si222(3O1) (The Grignard reagent was prepared under Schlenk conditions until workup) 4.14 g Magnesium turnings (170 mmol / 1.21 eq) were activated by dry stirring under vacuum, and 19 mL 1-bromo-3-methoxypropane (25.8 g / 169 mmol / 1.20 eq) in 100 mL dry THF were added dropwise over 2 h. After addition was complete, the reaction mixture was stirred at 40°C for 2 h, and 21.2 g Et_3SiCl (141 mmol / 1.00 eq) were added dropwise over 30 min. The mixture was heated to reflux for 16 h and then quenched by adding 60 mL half saturated aqueous NH_4Cl

solution. The phases were separated and the aqueous phase extracted with 100 mL pentane, setting aside the pentane phase. Both organic phases were dried over $MgSO_4$, and the solvent of the THF organic phased removed under reduced pressure using a rotary evaporator. The residue was taken up with the pentane organic phase, and the solution filtered through a Celite plug. Volatile components were removed under reduced pressure (1 mbar). The residue was purified by distillation (1-2 mbar, 70°C bath temperature), giving 23.7 g triethyl(3-methoxypropyl) silane (126 mmol / 89%). ^{1}H NMR (DMSO−d_6, 400 MHz, δ in ppm): 3.33 (s, 3H, O−$\mathbf{CH_3}$), 3.32 (t, $^3J_{H/H}$ = 6.9 Hz, 2H, $\mathbf{CH_2}$−O), 1.62-1.48 (m, 2H, $\mathbf{CH_2}$−CH_2−O), 0.91 (t, $^3J_{H/H}$ = 7.9 Hz, 9H, CH_2−$\mathbf{CH_3}$), 0.50 (q, $^3J_{H/H}$ = 8.0 Hz, 6H, $\mathbf{CH_2}$−CH_3), 0.49 (q, $^3J_{H/H}$ = 8.0 Hz, 2H, Si−$\mathbf{CH_2}$); ^{29}Si NMR ($CDCl_3$, 80 MHz, δ in ppm): 7.15 (s); ^{13}C{^{1}H} NMR (DMSO−d_6, 101 MHz, δ in ppm): 76.12 (s, $\mathbf{CH_2}$−O), 58.58 (s, O−$\mathbf{CH_3}$), 24.09 (s, $\mathbf{CH_2}$−CH_2−O), 7.55 (s, CH_2−$\mathbf{CH_3}$), 7.39 (s, Si−$\mathbf{CH_2}$), 3.37 (s, $\mathbf{CH_2}$−CH_3). Elemental analysis, using chromosorb to suppress evaporation: calculated for $C_{10}H_{24}OSi$: C, 63.76; H, 12.84; N, 0.00. Found C, 65.11; H, 13.77; N, 0.14.

[P2225][OAc] 6.02 g [P2225]Br (22.4 mmol / 1.00 eq) dissolved in 5 mL water were converted into aqueous [P2225]OH by slowly passing the solution over a strongly basic anion exchange resin (AmberLite IRN78 OH, SIGMA-ALDRICH, St. Louis, USA) and collecting all eluent with pH $\geq$ 7 (250 mL). Then, 1.34 g glacial acetic acid (22.4 mmol / 1.00 eq) were added, the solvent removed under reduced pressure using a rotary evaporator and the residue dried in high vacuum, giving 5.37 g triethyl(pentyl)phosphonium acetate (21.6 mmol / 96%). ^{1}H NMR (DMSO−d_6, 400 MHz, δ in ppm): 2.31 (dq, $^2J_{H/P}$ = 13.6 Hz, $^3J_{H/H}$ = 7.6 Hz, 6H, P−$\mathbf{CH_2}$−CH_3), 2.30-2.22 (m, 2H, P−$\mathbf{CH_2}$−CH_2), 1.48 (s, 3H, $\mathbf{CH_3}COO^-$), 1.53-1.40 (m, 2H, P−CH_2−$\mathbf{CH_2}$), 1.39-1.20 (m, 4H, $(\mathbf{CH_2})_2$−CH_3), 1.19 (dt, $^3J_{H/P}$ = 18.0 Hz, $^3J_{H/H}$ = 7.7 Hz, 9H, P−CH_2−$\mathbf{CH_3}$), 0.86 (t, $^3J_{H/H}$ = 7.1 Hz, 3H, CH_2−CH_2−$\mathbf{CH_3}$); ^{13}C{^{1}H} NMR (DMSO−d_6, 101 MHz, δ in ppm): 172.04 (s, $CH_3\mathbf{COO}^-$), 32.34 (d, $^3J_{H/P}$ = 15.2 Hz, $\mathbf{CH_2}$−CH_2−CH_3), 26.35 (s, $\mathbf{CH_3}COO^-$), 21.40 (s, CH_2−$\mathbf{CH_2}$−CH_3), 20.25 (d, $^2J_{C/P}$ = 4.4 Hz, P−CH_2−$\mathbf{CH_2}$), 16.39 (d, $^1J_{C/P}$ = 47.5 Hz, P−$\mathbf{CH_2}$−CH_2), 13.63 (s, CH_2−CH_2−$\mathbf{CH_3}$), 10.49 (d, $^1J_{C/P}$ = 48.7 Hz, P−$\mathbf{CH_2}$−CH_3), 5.25 (d, $^2J_{C/P}$ = 5.2 Hz, P−CH_2−$\mathbf{CH_3}$); ^{31}P{^{1}H} NMR (DMSO−d_6, 162 MHz, δ in ppm): 38.95 (s).

[P4441][OAc] 5 mL tributyl phosphine (4.02 g / 19.8 mmol / 1.00 eq), 5 mL MeOH, and 3 mL dimethyl carbonate (3.21 g / 35.6 mmol / 1.80 eq) were added to a 20 mL pressure tube (Ace Glass, Vineland, USA) with silicone seal and heated to 110°C for 24 h. After

that, 1.20 g glacial acetic acid (19.9 mmol / 1.01 eq) were added, and the solvent removed under reduced pressure using a rotary evaporator. Drying in high vacuum gave 5.63 g tributylmethylphosphonium acetate (20.3 mmol / quantitative yield). The product contains ≈ 3% of the secondary phosphonium species as impurity, as determined by $^{31}P\{^1H\}$ NMR. ^1H NMR (DMSO−d_6, 400 MHz, δ in ppm): 2.29-2.13 (m, 6H, P−$\mathbf{CH_2}$), 1.84 (d, $^2J_{H/P}$ = 14.2 Hz, 3H, P−CH_3), 1.50 (s, 3H, $\mathbf{CH_3}COO^-$), 1.50-1.30 (m, 12H, ($\mathbf{CH_2}$)$_2$−CH_3), 0.90 (t, $^3J_{H/H}$ = 7.1 Hz, 9H, CH_2−$\mathbf{CH_3}$); $^{13}C\{^1H\}$ NMR (DMSO−d_6, 101 MHz, δ in ppm): 172.09 (s, $CH_3\mathbf{COO}^-$), 26.22 (s, $\mathbf{CH_3}COO^-$), 23.32 (d, $^3J_{H/P}$ = 15.9 Hz, $\mathbf{CH_2}$−CH_3), 22.63 (d, $^2J_{C/P}$ = 4.3 Hz, P−CH_2−$\mathbf{CH_2}$), 18.80 (d, $^1J_{C/P}$ = 49.2 Hz, P−$\mathbf{CH_2}$−CH_2), 13.26 (s, CH_2−$\mathbf{CH_3}$), 3.00 (d, $^1J_{C/P}$ = 51.2 Hz, P−$\mathbf{CH_3}$); $^{31}P\{^1H\}$ NMR (DMSO−d_6, 162 MHz, δ in ppm): 32.61 (s).

[P2225][**Br**]: 6.30 mL of triethyl phosphine (5.05 g / 42.7 mmol / 1.00 eq) were dissolved in 200 mL of dry, degassed acetonitrile under argon and 7.41 mL of 1-bromo-pentane (9.02 g / 59.8 mmol / 1.40 eq) were added dropwise. The obtained clear, homogeneous solution was stirred for 4 days at ambient temperature before the solvent and excess of alkyl bromide were removed by rotary evaporation and the residue dried in high vacuum to obtain 11.3 g of the triethyl pentyl phosphonium bromide (41.8 mmol/ 99% yield) as white solid. ^1H NMR (DMSO−d_6, 400 MHz, δ in ppm): 2.32-2.16 (m, 8H, P−$\mathbf{CH_2}$), 1.56-1.42 (m, 2H, P−CH_2−$\mathbf{CH_2}$), 1.42-1.26 (m, 4H, P−(CH_2)$_2$−($\mathbf{CH_2}$)$_2$), 1.12 (dt, $^3J_{H/P}$ = 17.9 Hz, $^3J_{H/H}$ = 7.7 Hz, 9H, P−CH_2−$\mathbf{CH_3}$), 0.88 (t, $^3J_{H/H}$ = 7.1 Hz, 3H, P−(CH_2)$_4$−$\mathbf{CH_3}$); $^{13}C\{^1H\}$ NMR (DMSO−d_6, 101 MHz, δ in ppm): 32.2 (d, $^4J_{H/P}$ = 14.8 Hz, P−(CH_2)$_2$−$\mathbf{CH_2}$), 21.34 (s, P−(CH_2)$_3$−$\mathbf{CH_2}$), 20.20 (d, $^3J_{H/P}$ = 4.4 Hz, P−CH_2−$\mathbf{CH_2}$), 16.43 (d, $^2J_{H/P}$ = 47.4 Hz, P−$\mathbf{CH_2}$)−(CH_2)$_4$−H), 13.63 (s, P−(CH_2)$_4$−$\mathbf{CH_3}$), 10.59 (d, $^2J_{H/P}$ = 48.8 Hz, P−$\mathbf{CH_2}$)−CH_3), 5.22 (d, $^3J_{H/P}$ = 5.3 Hz, P−CH_2−$\mathbf{CH_3}$); $^{31}P\{^1H\}$ NMR (DMSO−d_6, 162 MHz, δ in ppm): 38.94 (s).

[P2225][**$O_2C(CH_2)_4H$**]: 7.62 g of [P2225][Br] (28.3 mmol / 1.00 eq) were dissolved in approximately 25 mL deionized water and the obtained homogeneous solution passed through an Amberlyst A-27 anion exchange resign. The obtained hydroxide solution of the phosphonium cation was immediately neutralized with a dilute aqueous solution of valeric acid (monitored using a digital pH meter). The neutralized solution was dried using an rotary evaporator and the residue dried on a Schlenk line for two days with stirring. The product was obtained as a colorless liquid which solidified under pronounced shearing (8.14 g/ 28.0 mmol/ 99% yield). The DSC trace is shown in Figure 11. ^1H NMR ($CDCl_3$, 400 MHz, δ in ppm): 2.46 (dq, 6H,

$^2J_{H/P}$ = 13.3 Hz, $^3J_{H/H}$ = 7.7 Hz, P−CH$_2$−**CH$_3$**), 2.35-2.22 (m, 2H, P−CH$_2$−**CH$_2$**), 2.07 (t, $^3J_{H/H}$ = 8.0 Hz, 2H, O$_2$C−CH$_2$), 1.56-1.41 (m, 4H, P−CH$_2$−**CH$_2$** + O$_2$C−CH$_2$−**CH$_2$**), 1.41-1.32 (m, 2H, O$_2$C−(CH$_2$)$_2$−**CH$_2$**), 1.31-1.11 (m, 13H, P−(CH$_2$)$_2$−(**CH$_2$**)$_2$ + P−CH$_2$−**CH$_3$**), 0.85-0.76 (m, 6H, P−(CH$_2$)$_5$−**CH$_3$** + O$_2$C−(CH$_2$)$_4$−**CH$_3$**); ^{13}C{^{1}H} NMR (CDCl$_3$, 101 MHz, δ in ppm): 179.21 (s, O$_2$C), 38.8 (s, O$_2$C−**CH$_2$**), 32.6 (d, $^4J_{H/P}$ = 14.5 Hz, P−(CH$_2$)$_2$−**CH$_2$**), 29.2 (s, O$_2$C−CH$_2$−**CH$_2$**), 22.81 (s, O$_2$C−(CH$_2$)$_2$−**CH$_2$**), 21.73 (s, P−(CH$_2$)$_3$−**CH$_2$**), 21.08 (d, $^3J_{H/P}$ = 4.8 Hz, P−CH$_2$−**CH$_2$**), 17.40 (d, $^2J_{H/P}$ = 47.0 Hz, P−**CH$_2$**)−(CH$_2$)$_4$−H), 13.85 (s, P−(CH$_2$)$_4$−**CH$_3$**), 13.42 (s, O$_2$C−(CH$_2$)$_3$−**CH$_3$**) 10.54 (d, $^2J_{H/P}$ = 48.7 Hz, P−**CH$_2$**)−CH$_3$), 5.69 (d, $^3J_{H/P}$ = 5.6 Hz, P−CH$_2$−**CH$_3$**); ^{31}P{^{1}H} NMR (DMSO−d$_6$, 162 MHz, δ in ppm): 38.25 (s).

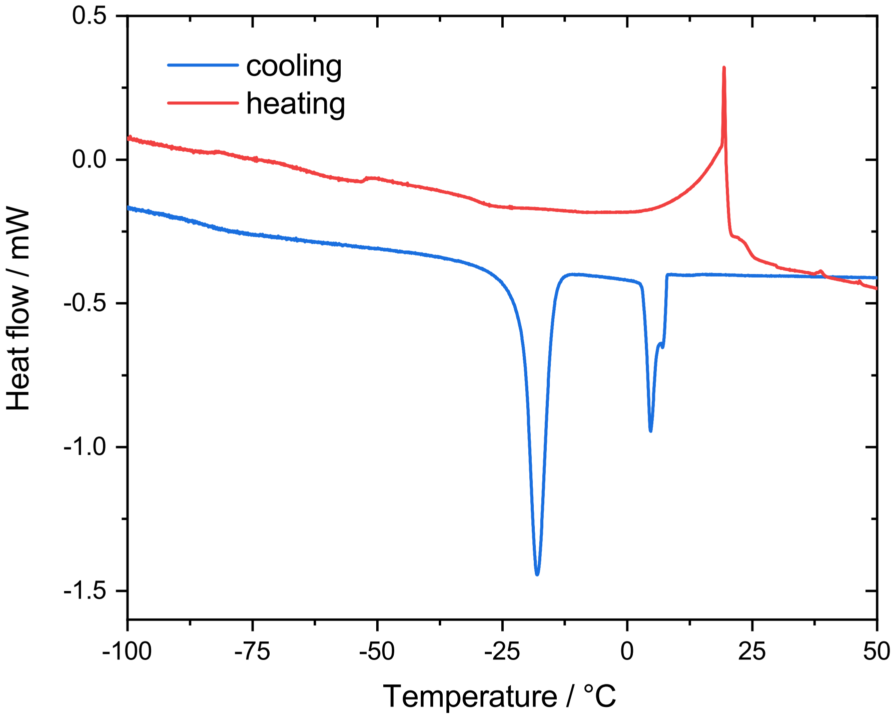

Figure 11: DSC trace of the ionic liquid P2225Valerinate

References

(1) G. Morrison and J. E. Lind, *The Journal of Chemical Physics*, 1968, **49**, 5310–5316.

(2) E. R. López, A. S. Pensado, M. J. P. Comuñas, A. A. H. Pádua, J. Fernández and K. R. Harris, *The Journal of Chemical Physics*, 2011, **134**, 144507.

(3) K. R. Harris and M. Kanakubo, *Faraday Discussions*, 2012, **154**, 425–438.

(4) S.-W. Park, S. Kim and Y. Jung, *Physical Chemistry Chemical Physics*, 2015, **17**, 29281–29292.

(5) H. Shirota and E. W. Castner, *The Journal of Physical Chemistry A*, 2005, **109**, 9388–9392.

(6) S. Grimme, J. Antony, S. Ehrlich and H. Krieg, *The Journal of Chemical Physics*, 2010, **132**, 154104.

(7) S. Grimme, S. Ehrlich and L. Goerigk, *Journal of Computational Chemistry*, 2011, **32**, 1456–1465.

(8) M. J. Frisch and G. Trucks, *Gaussian 09*, Wallingford CT, 2016.

(9) F. Philippi, A. Quinten, D. Rauber, M. Springborg and R. Hempelmann, *The Journal of Physical Chemistry A*, 2019, **123**, 4188–4200.

(10) T. Lu and F. Chen, *Journal of Computational Chemistry*, 2012, **33**, 580–592.

(11) T. Lu and F. Chen, *Journal of Molecular Graphics and Modelling*, 2012, **38**, 314–323.

(12) D. Rauber, F. Philippi, J. Zapp, G. Kickelbick, H. Natter and R. Hempelmann, *RSC Advances*, 2018, **8**, 41639–41650.

(13) A. E. Taouil, F. Lallemand, J.-M. Melot, J. Husson, J.-Y. Hihn and B. Lakard, *Synthetic Metals*, 2010, **160**, 1073–1080.

(14) C. Li, Z. Zhang, J. Wu, D. Li and W. Hou, *Polymer International*, 2014, **63**, 1875–1880.

(15) A. Kreyes, A. Mourran, Z. Hong, J. Wang, M. Möller, F. Gholamrezaie, W. S. C. Roelofs, D. M. de Leeuw and U. Ziener, *Chemistry of Materials*, 2013, **25**, 2128–2136.

(16) D. W. Keefer, H. Gou, Q. Wang, A. Purdy, A. Epshteyn, S. J. Juhl, G. D. Cody, J. Badding and T. A. Strobel, *The Journal of Physical Chemistry A*, 2018, **122**, 2858–2863.

(17) E. Mayer, *Monatshefte für Chemie*, 1969, **100**, 462–468.

(18) D. Gardiner and E. Mayer, *Journal of Molecular Structure*, 1973, **16**, 173–178.
